# Atomically dispersed nickel–nitrogen–sulfur species anchored on porous carbon nanosheets for efficient water oxidation

**DOI:** 10.1038/s41467-019-09394-5

**Published:** 2019-03-27

**Authors:** Yang Hou, Ming Qiu, Min Gyu Kim, Pan Liu, Gyutae Nam, Tao Zhang, Xiaodong Zhuang, Bin Yang, Jaephil Cho, Mingwei Chen, Chris Yuan, Lecheng Lei, Xinliang Feng

**Affiliations:** 1 Key Laboratory of Biomass Chemical Engineering of Ministry of Education, College of Chemical and Biological Engineering, Zhejiang University, 310027 Hangzhou, China; 2 Center for Advancing Electronics Dresden (cfaed) & Department of Chemistry and Food Chemistry, Technische Universitaet Dresden, 01062 Dresden, Germany; 3 Institute of Nanoscience and Nanotechnology, College of Physical Science and Technology, Central China Normal University, 430079 Wuhan, China; 4 Beamline Division, Pohang Accelerator Laboratory, Pohang, Kyungbuk 37673 Republic of Korea; 5 WPI Advanced Institute for Materials Research, Tohoku University, Sendai, 980-8577 Japan; 6 CREST, JST, 4-1-8 Honcho Kawaguchi, Saitama, 332-0012 Japan; 7 Department of Energy Engineering, School of Energy and Chemical Engineering, Ulsan National Institute of Science and Technology (UNIST), Ulsan, 44919 Republic of Korea; 8 Department of Mechanical and Aerospace Engineering, Case Western Reserve University, Cleveland, OH 44106 USA

## Abstract

Developing low-cost electrocatalysts to replace precious Ir-based materials is key for oxygen evolution reaction (OER). Here, we report atomically dispersed nickel coordinated with nitrogen and sulfur species in porous carbon nanosheets as an electrocatalyst exhibiting excellent activity and durability for OER with a low overpotential of 1.51 V at 10 mA cm^−2^ and a small Tafel slope of 45 mV dec^−1^ in alkaline media. Such electrocatalyst represents the best among all reported transition metal- and/or heteroatom-doped carbon electrocatalysts and is even superior to benchmark Ir/C. Theoretical and experimental results demonstrate that the well-dispersed molecular S|NiN_x_ species act as active sites for catalyzing OER. The atomic structure of S|NiN_x_ centers in the carbon matrix is clearly disclosed by aberration-corrected scanning transmission electron microscopy and synchrotron radiation X-ray absorption spectroscopy together with computational simulations. An integrated photoanode of nanocarbon on a Fe_2_O_3_ nanosheet array enables highly active solar-driven oxygen production.

## Abstract (web summary)

Water oxidation is considered the bottleneck reaction for light-driven water splitting due to the sluggish kinetics and poor stability. Here, authors show metal- and heteroatom-doped carbons as effective catalysts for both electrochemical and photoelectrochemical water splitting.

## Introduction

Solar-driven photoelectrochemical (PEC) water splitting is considered as one of the most promising strategies for producing renewable energy carriers using sunlight and water^1,2^. In addition to a light absorber that generates photoexcited electron-hole pairs, the PEC system requires an electrocatalyst to improve the kinetic barriers for the surface oxygen evolution reaction (OER)^3–5^. Currently, Ir/Ru-based materials are regarded as state-of-the-art electrocatalysts for OER, but their high cost and scarcity seriously hinder their widespread applications. Motivated by this challenge, significant efforts have been devoted to developing inexpensive non-precious metal OER electrocatalysts, such as transition metal (TM) oxides, hydroxides, phosphates, nitrides^6–16^, and perovskite oxides^10,13,17^. Ideal OER electrocatalysts for PEC water splitting require not only small overpotentials and low costs for OER but also stable electrical contacts with the photoelectrode^18^.

Recently, nanocarbon-based hybrids, especially TM–N_*x*_-doped carbon materials with charge polarization and asymmetric electron spin density, have attracted growing attention as promising OER electrocatalysts due to their tunable electronic structure and high electrical conductivity^19^. Despite certain progress^19–21^, the application of TM–N_*x*_-doped nanocarbons in OER electrocatalysis is still in infancy, and their catalytic performance is far from satisfactory. To improve the overall catalytic activity, more efforts should be devoted to clarify the active sites of TM–N_*x*_-doped nanocarbons involved in the OER process for deciphering their actual catalytic mechanisms. With the help of density functional theory (DFT) calculations, the strong similarities between the active metal centers of synthetic nickel (II) porphyrin complex^22^ and reduced coordination environments of biological enzymes with molecular TM/N moiety^23,24^ suggest that the favorable catalytic sites might take the form of a tetracoordinate planar structure of the TM bridged by four N atoms^25^, labeled as coordinated TM–N_4_ architecture. Further theoretic investigations have demonstrated that the TM atom shows a high-reaction free energy for the adsorption of intermediate products owing to the strong electronegativity of neighboring N atoms, which increases the potential barriers in the reaction processes^26^. Thus, the TM’s electron-donating character can be modified by introducing proper foreign atoms^27^, which will reduce the potential barriers and help to improve the intrinsic catalytic activity of TM–N_*x*_-doped carbon. Given that the electronegativity of S atom is weaker than that of N atom, we predicted that the partial displacement of N with S atoms in coordinated TM–N_*x*_ can greatly influence its electronic structure and, thus improve the OER kinetics.

Herein, we report a nanocarbon OER electrocatalyst composed of atomically disperse S|NiN_*x*_ species embedded in porous carbon (PC) nanosheets (denoted S|NiN_*x*_−PC), which are synthesized by the pyrolysis of ternary dicyandiamide–thiophene–nickel salt nanocomposites grown on electrochemically exfoliated graphene (EG) foil at 900 °C in an Ar atmosphere, followed by an acid leaching treatment. The achieved S|NiN_*x*_−PC/EG nanosheets delivers a high OER performance with a low overpotential of 1.51 V at 10 mA cm^−2^ and a small Tafel slope of 45 mV dec^−1^ and are superior to all reported transition metal- and/or heteroatom-doped carbon catalysts and even the commercial Ir/C benchmark catalyst (1.59 V at 10 mA cm^−2^) in alkaline media. Experimental observations and computational studies reveal that the excellent OER performance originates from the formation of atomically isolated Ni atoms coordinated with three N atoms and one S atom in the carbon matrix, which can create sufficient localized reactive sites by modifying the local charge distribution on the carbon surface and reducing the potential barriers of the elementary reactions, thereby boosting its OER kinetics. The S|NiN_*x*_ species behave as active centers, as identified by synchrotron radiation X-ray absorption spectroscopy and spherical aberration-corrected electron microscopy (HAADF-STEM). Moreover, when the achieved nanocarbon is further integrated into the Fe_2_O_3_ nanorod array (Fe_2_O_3_-NA) for solar water oxidation, an AM 1.5G photocurrent density of 1.58 mA cm^−2^ at 1.23 V is delivered, outperforming those reported for other Fe_2_O_3_-based inorganic photoanodes.

## Results

### Electrocatalyst synthesis and characterizations

The synthetic procedure of the S|NiN_*x*_−PC/EG nanosheets is illustrated in Supplementary Fig. 1. A ternary supramolecular composite (TSC) was first obtained by the cooperative assembly of dicyandiamide, thiophene, and NiCl_2_ on the surface of EG foil under hydrothermal conditions. Fourier transform infrared spectra and liquid-state ^1^H nuclear magnetic resonance spectroscopy verified the successful coordination of thiophene with dicyandiamide/Ni^2+^ (Supplementary Figs. 2 and 3), resulting in the formation of TSC, which was further supported by a color difference between the products (Supplementary Fig. 4). Subsequent pyrolysis at 900 °C under an Ar atmosphere and acid leaching treatment converted the TSC/EG precursor into S|NiN_*x*_−PC/EG (Supplementary Fig. 5). During the pyrolysis process, the continuous decomposition of TSC was accompanied by releasing N- and S-containing gases^28,29^, which generated porous structures.

**Fig. 1:**
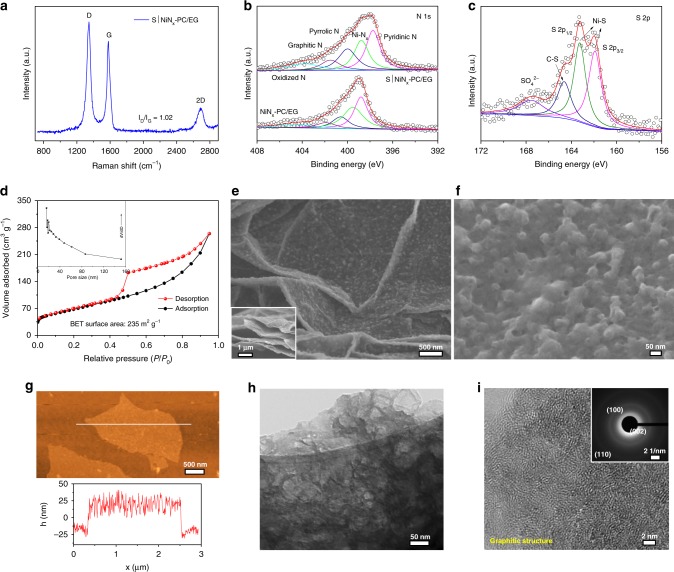
Morphological and structural characterizations. **a** Raman spectrum, **b**, **c** High-resolution N 1s and S 2p XPS spectra, **d** N_2_ adsorption isotherm and corresponding pore-size distributions (inset), **e**, **f** FESEM images, **g** AFM image, **h**, **i** TEM and HRTEM images of S|NiN_*x*_−PC/EG. Inset in **i**: SAED pattern of S|NiN_*x*_−PC/EG. Data for NiN_*x*_−PC/EG is also shown

X-ray diffraction (XRD) patterns and Raman spectra confirm the formation of graphitic carbon in the S|NiN_*x*_−PC/EG during pyrolysis (Fig. 1a and Supplementary Figs. 6 and 7)^30^. X-ray photoelectron spectroscopy (XPS) reveals that the S|NiN_*x*_−PC/EG is mainly consisted of Ni, N, S, C, and O elements (Supplementary Fig. 8). The high-resolution Ni 2p spectra of S|NiN_*x*_−PC/EG display the binding energies of the Ni 2p_3/2_ and Ni 2p_1/2_ peaks at 854.9 eV and 872.3 eV with two satellite peaks at 861.2 eV and 879.8 eV, respectively, which are characteristic of Ni^2+^ and Ni^3+^ (Supplementary Fig. 9)^31^. The high-resolution N 1s spectrum is deconvoluted into five types of N species (Fig. 1b), which correspond to pyridinic N (397.8 eV), Ni–N_*x*_ (398.8 eV), pyrrolic N (400.0 eV), graphitic N (401.5 eV), and oxidized N (403.9 eV)^32^. Clear shifts in the binding energy of the Ni 2p and N 1s peaks of S|NiN_*x*_−PC/EG compared to those of NiN_*x*_−PC/EG are observed (Fig. 1b and Supplementary Fig. 10), indicating that the S atoms likely coordinate with the Ni atoms by partial replacement of the N atoms to form Ni–S_*x*_ sites^19^, which thus optimize the local electronic structure of S|NiN_*x*_−PC/EG. The high-resolution S 2p XPS spectrum of S|NiN_*x*_−PC/EG confirms the existence of Ni–S and C–S bonds (Fig. 1c)^33,34^. The bonds between C and N or S (C–N/C–S) are also supported by the peak centered at 285.3 eV in the C 1s spectrum (Supplementary Fig. 8). The Nitrogen adsorption−desorption isotherm displays a mesoporous feature of S|NiN_*x*_−PC/EG with a Brunauer−Emmett−Teller (BET) surface area of 235 m^2 ^g^−1^, a pore-size distribution centered at ∼18 nm and a total pore volume of 0.41 cm^3^ g^−1^ (Fig. 1d). In addition, the S|NiN_*x*_−PC/EG is highly hydrophilic with a small contact angle of 35.4°, which enables the electrolyte to access the active surface (Supplementary Fig. 11).

Field-emission scanning electron microscopy (FESEM) images of S|NiN_*x*_−PC/EG show a two-dimensional (2D) sheet-like morphology with a lateral size of up to several micrometers and the appearance of some observable mesopores (Fig. 1e, f and Supplementary Fig. 12). Atomic force microscopy (AFM) reveals that the thickness of the S|NiN_*x*_−PC/EG nanosheets is ~32 nm (Fig. 1g). Elemental mapping spectroscopy confirms that the S|NiN_*x*_−PC/EG is composed of Ni, N, S, C, and O elements (Supplementary Fig. 13). Further transmission electron microscopy (TEM) and high-resolution TEM (HRTEM) analysis manifest the partially graphitized nature and highly porous structure of these 2D S|NiN_*x*_−PC/EG nanosheets (Fig. 1h, i).

### Electrocatalytic OER

**Fig. 2:**
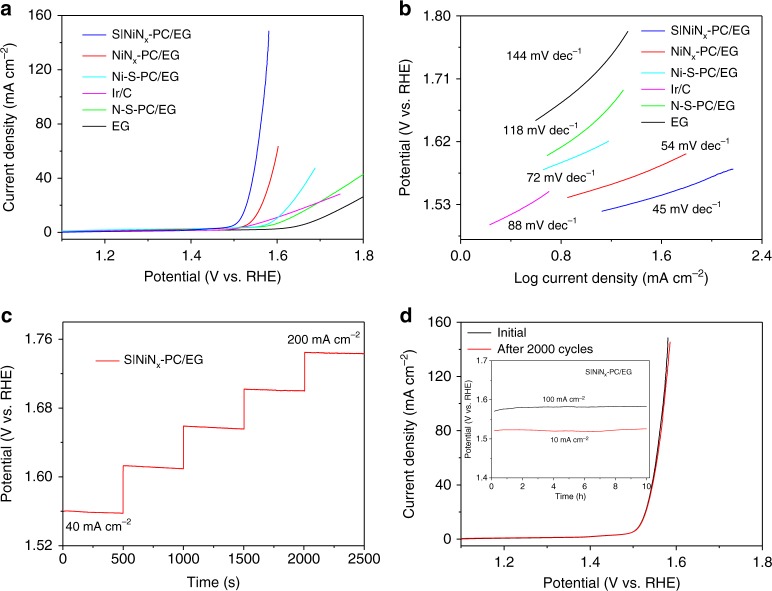
Electrocatalytic OER performance. **a** Polarization curves of EG, NiN_*x*_−PC/EG, Ni-S−PC/EG, N-S−PC/EG, S|NiN_*x*_−PC/EG, and Ir/C for OER. **b** The corresponding Tafel plots. **c** Multi-current electrochemical process of S|NiN_*x*_−PC/EG. **d** Polarization curves of S|NiN_*x*_−PC/EG before and after 2000 cycles. Inset: Chronopotentiometry curves of S|NiN_*x*_−PC/EG under different current densities of 10 and 100 mA cm^−2^. All experiments were carried out in 1.0 M KOH

The electrocatalytic activity of S|NiN_*x*_−PC/EG toward OER was investigated in 1.0 M KOH. For comparison, NiN_*x*_−PC/EG, Ni/S co-doped PC/EG (Ni-S−PC/EG), N/S co-doped PC/EG (N-S−PC/EG), and EG were also prepared under similar conditions. Among them, S|NiN_*x*_−PC/EG exhibited the best OER performance with the smallest onset potential of 1.50 V and the highest catalytic current density across the entire potential range (Fig. 2a), which was superior to the onset potentials of NiN_*x*_−PC/EG (1.53 V), Ni-S−PC/EG (1.57 V), N-S−PC/EG (1.58 V), and EG (1.60 V), revealing the pivotal effect of the S|NiN_*x*_ complex doped in the carbon matrix for OER. Moreover, the S|NiN_*x*_−PC/EG afforded current densities of 10 and 100 mA cm^−2^ at overpotentials of 1.51 and 1.56 V, respectively. The achieved overpotentials are the lowest among all heteroatom- and/or transition metal-doped carbon electrocatalysts for OER reported thus far (Supplementary Table 1), and they even surpass the state-of-the-art commercial Ir/C catalyst (1.59 V at 10 mA cm^−2^). The mass activity of S|NiN_*x*_−PC/EG was 941.8 mA mg^−1^ at 1.58 V, which is ~16.2 times higher than that of commercial Ir/C (58.1 mA mg^−1^). Assuming that all the Ni sites were electrochemically active in the OER process, the calculated turnover frequency (TOF) of S|NiN_*x*_−PC/EG reached 10.9 s^−1^ (Supplementary Fig. 14 and Supplementary Table 2). We further explored the influence of the pyrolysis temperature (700–1000 °C) and molar ratio of dicyandiamide:thiophene:Ni^2+^. The highest OER activity was achieved with a pyrolysis temperature at 900 °C and molar ratio of 10:10:1 (Supplementary Figs. 15–18). The corresponding Tafel slope of S|NiN_*x*_−PC/EG was measured as 45 mV dec^−1^ (Fig. 2b), which is smaller than that of the Ir/C catalyst (88 mV dec^−1^), suggesting its favorable catalytic kinetics for OER. The electrochemical impedance spectra (EIS) revealed that S|NiN_*x*_−PC/EG possessed the smallest charge-transfer resistance among all four samples (Supplementary Fig. 19), further justifying the promoted OER kinetics^35^.

The multi-step chronopotentiometric curve showed that at the start of 40 mA cm^−2^ (Fig. 2c), the potential immediately leveled off at 1.55 V and remained unchanged for the remaining 500 s. The other steps also showed similar results up to 200 mA cm^−2^, implying that the outstanding mass transport property and mechanical robustness of S|NiN_*x*_−PC/EG. The polarization curve of S|NiN_*x*_−PC/EG exhibited a negligible loss even after 2000 cycles, indicating its high electrochemical stability (Fig. 2d). Additionally, the durability tests revealed that S|NiN_*x*_−PC/EG retained its catalytic activity over 10 h at both 10 and 100 mA cm^−2^ (inset of Fig. 2d), which is superior to that of NiN_*x*_−PC/EG (Supplementary Figs. 20–22).

### Understanding the active sites

**Fig. 3:**
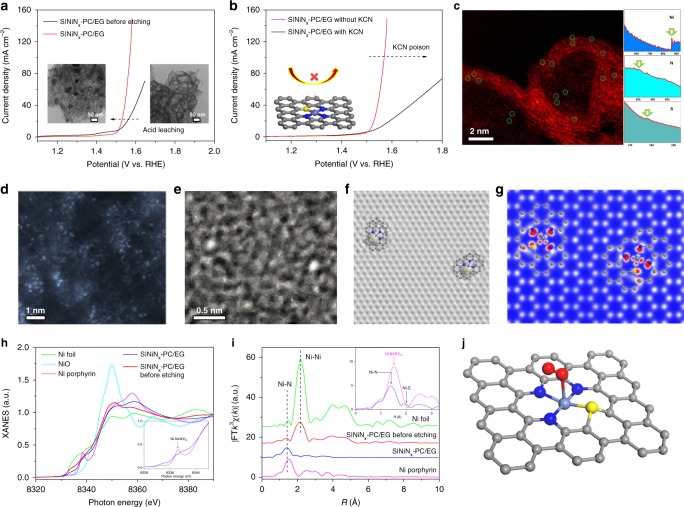
Understanding the structure of active sites. **a** Comparison of the OER activity of S|NiN_*x*_−PC/EG and S|NiN_*x*_−PC/EG before etching. Insets are TEM images showing that the Ni nanoparticles were removed by acid etching treatment. **b** Polarization curves of S|NiN_*x*_−PC/EG with and without 10 mM KCN, indicating that CN^−^ ions strongly poison the S|NiN_*x*_−PC/EG. Insets: illustrations of S|NiN_*x*_ centers blocked by the CN^−^ ions. **c** HAADF-STEM image of S|NiN_*x*_−PC/EG and corresponding electron energy loss spectroscopy atomic spectra of Ni, N, and S elements from the bright dots, as shown by the green circle arrow in **c**. **d**, **e** Atomic-resolution HAADF-STEM images of S|NiN_*x*_−PC/EG. **f**, **g** Simulated HRTEM and STM images for **e**. **h** Ni K-edge XANES spectrum and **i** Ni K-edge k3-weighted EXAFS spectrum of S|NiN_*x*_−PC/EG; data for the Ni foil, NiO, Ni porphyrin, and S|NiN_*x*_−PC/EG before etching are also shown. The insets are the magnified images. **j** Schematic structural model for S|NiN_*x*_−PC. The steel blue, blue, yellow, gray, and red spheres represent Ni, N, S, C, and O atoms, respectively

Control experiments demonstrate that the S|NiN_*x*_−PC/EG prepared without acid leaching led to a decrease in activity (Fig. 3a), highlighting that the metallic nickel or nickel oxide nanoparticles formed during pyrolysis are inactive or might block the active site for OER. Acid leaching eliminated the inactive Ni species and increased the exposure of S|NiN_*x*_ species, as confirmed by the HRTEM analysis and N_2_ sorption studies (Fig. 3a and Supplementary Fig. 23). These results, coupled with the XPS analysis, energy-dispersive X-ray spectrometer (EDX) mapping, and the influence of metal ions (Co^2+^, Fe^3+^, and Ni^2+^) on the OER activity (Supplementary Fig. 24), identify the crucial role of well-dispersed S|NiN_*x*_ species as active centers towards OER. To confirm the presence of NiN_*x*_ centers, cyanide poisoning experiments of S|NiN_*x*_−PC/EG and NiN_*x*_−PC/EG were conducted. After treatment with potassium cyanide, both samples suffered from decreased activity (Fig. 3b and Supplementary Fig. 25), which undoubtedly indicated that the NiN_*x*_ active sites with S-doping were the origin of the OER activity for S|NiN_*x*_−PC/EG^36,37^. Atomic-resolution high-angle annular dark-field scanning transmission electron microscope (HAADF-STEM) images disclose that numerous atomically dispersed bright spots marked with green cycles, corresponding to heavier Ni atoms, are distinguished in the porous carbon frameworks (Fig. 3c). The enlarged view of the selected region and atomic electron energy loss spectroscopy (EELS) of the bright dots (Fig. 3c–e) demonstrates that each Ni atom is coordinated by N and S elements and further hybridized in the carbon matrix. This observation correlates well with the DFT–simulated STEM and scanning tunneling microscopy (STM) images, showing that the Ni–N–S center is embedded in the carbon lattice, forming stable bonds with neighboring carbon atoms (Fig. 3f, g). The HAADF-STEM images and corresponding EDX mapping of S|NiN_*x*_−PC/EG further demonstrate that the Ni, N, and S atoms are homogenously distributed throughout the whole sample (Supplementary Fig. 26). The Ni content of S|NiN_*x*_−PC/EG is 0.2 wt.%, as measured by inductively coupled plasma–optical emission spectrometry.

To further probe the chemical state and local coordination structure of the Ni atoms in S|NiN_*x*_−PC/EG, X-ray absorption near-edge structure (XANES) and extended X-ray absorption fine structure (EXAFS) spectroscopy measurements were performed (Fig. 3h, i and Supplementary Figs. 27 and 28). As shown in Fig. 3h, a distinct shoulder peak at 8338 eV in the inset XANES figure (with arrow) presents a characteristic peak featuring the Ni–N_4_ square planar D_4h_ symmetry in the reference Ni porphyrin^38^. The S|NiN_*x*_−PC/EG shows a smaller change in the shoulder peak than that of Ni porphyrin, indicating that a distorted Ni-[N/S]_4_ bonding environment deviated from the ideal square planar geometry, which can be supported by the corresponding radial distribution function (RDF) of the Fourier-transformed (FT) EXAFS spectra (Fig. 3i). Compared to the RDF of the reference Ni porphyrin, which shows a symmetric FT peak (Ni–N_4_) in the inset RDF figure, S|NiN_*x*_−PC/EG has a well-separated FT peak featuring shorter and longer bond distances of 1.85(3)Å and 2.33(1)&Aring, which correspond to Ni–N and Ni–S, respectively. With the EXAFS fitting process, the coordination numbers of Ni–N and Ni–S in S|NiN_*x*_−PC/EG are calculated to be 2.8(2) and 0.8(3), compared to those of the Ni–N_4_ in Ni porphyrin (Fig. 3j). The substitution of larger sulfur for one nitrogen in the D_4h_ local structure results in the shortening of the Ni–N bond distance and a slight local distortion from the ideal square planar symmetry^39^.

### Theoretical investigation

To rationalize the four-electron reaction mechanism and high electrocatalytic activity of S|NiN_*x*_−PC/EG for OER, the correlative theoretical calculations were performed through DFT. A large amount of possible N–S, Ni–S, Ni–N_4_, Ni–N_3_S, Ni–N_2_S_2_, and Ni–NS_3_ models were judiciously constructed (partial models can be found in Supplementary Figs. 29–34). In the calculations, the N–S and Ni–S models are the N/S and Ni/S co-doped graphene structures, respectively; the Ni–N_4_, Ni–N_3_S, Ni–N_2_S_2_, and Ni–NS_3_ models are the Ni–N_4_-, Ni–N_3_S-, Ni–N_2_S_2_-, and Ni–NS_3_-doped graphene structures. From the formation energy calculations, the Ni–N_4_ and Ni–N_3_S models have the lowest formation energy values and are the most stable structures among these models. Considering the correction of zero point energy, the formation energies for Ni–N, Ni–S, and N–S models are higher than that of Ni–N_3_S model, which indicates that the Ni–N_3_S model can be more thermally stabilized than Ni–N, Ni–S, and N–S models. Each value of the overpotential *η* for the catalytically active sites on all the models is calculated to further evaluate the catalytic activities of different electrocatalysts. For the N–S model, the S and C atoms neighboring the N atom, which are typical electron donors^40^, possess high-potential barriers for the rate-limiting step in OER. Meanwhile, from our calculations, the OER pathways on S in the Ni–N_3_S models and some C atoms, which are neighboring the N atoms in the Ni–N, Ni–N_4_ and Ni–N_3_S models, also show high free-energy values in the third steps (OOH* generation) because of edge effects^41^. These potential barriers prevent them from exhibiting better catalytic activities than the Ni atoms of Ni–N_4_ and Ni–N_3_S in these models. Consequently, the Ni atoms in the Ni-doped models (such as the Ni–N, Ni–N_4_, and Ni–N_3_S models) are confirmed to be the most active catalytic sites for OER^42^.

**Fig. 4:**
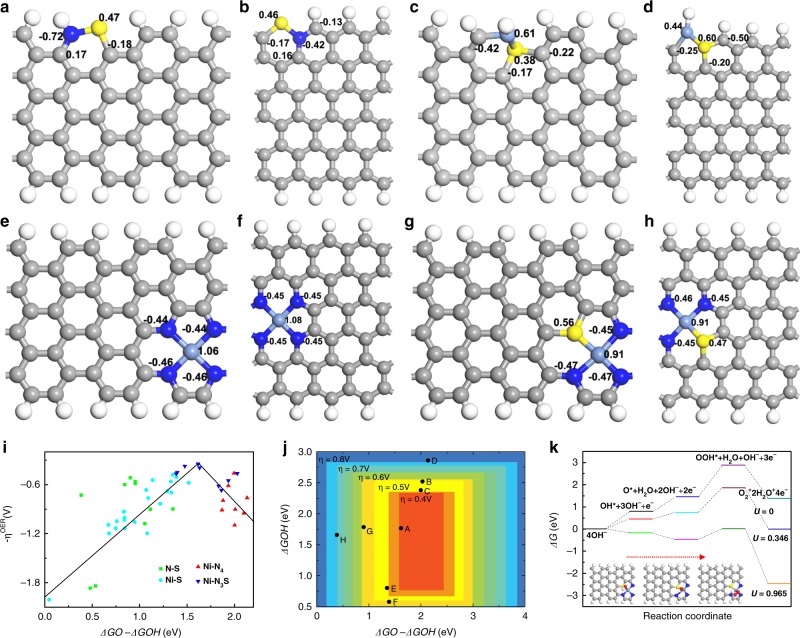
Theoretical calculations. Population distributions for the DFT-calculated representative models: **a** N−S co-doped armchair nanoribbon, **b** N−S co-doped zigzag nanoribbon, **c** Ni−S co-doped armchair nanoribbon, **d** Ni−S co-doped zigzag nanoribbon, **e** Ni−N_4_-doped armchair nanoribbon, **f** Ni−N_4_-doped zigzag nanoribbon, **g** Ni−N_3_S-doped armchair nanoribbon, **h** Ni−N_3_S-doped zigzag nanoribbon. **i** OER volcano plot of the overpotential *η* vs. the difference between the adsorption free energy of O* and OH* for the N−S, Ni−S, Ni−N_4_, and Ni−N_3_S models. **j** Adsorption free energy of OH* vs. the difference between the adsorption free energy of O* and OH* for the N−S, Ni−S, Ni−N_4_, and Ni−N_3_S models. **k** Schematic free-energy profile for the OER pathway on the Ni−N_3_S model in alkaline media

**Fig. 5:**
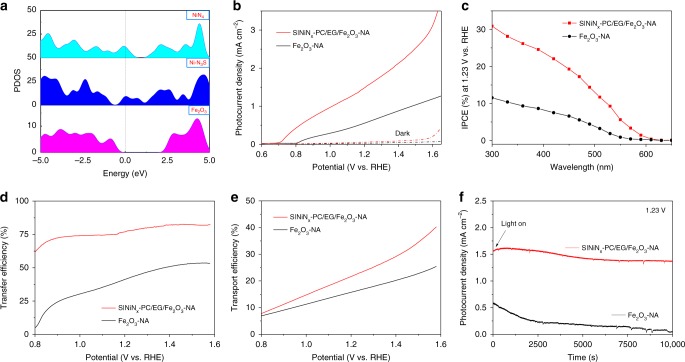
PEC-OER performance. **a** DFT-calculated projected density-of-states for the Ni−N_4_, Ni−N_3_S, and Fe_2_O_3_ models. **b** Variation in the photocurrent density vs. applied voltage for Fe_2_O_3_-NA and S|NiN_*x*_−PC/EG/Fe_2_O_3_-NA under dark and AM 1.5G irradiation. **c** IPCE spectra of Fe_2_O_3_-NA and S|NiN_*x*_−PC/EG/Fe_2_O_3_-NA under AM 1.5G irradiation. **d**, **e** Charge-transfer efficiencies and charge transport efficiencies of Fe_2_O_3_-NA and S|NiN_*x*_−PC/EG/Fe_2_O_3_-NA. **f** Transient photocurrent responses of Fe_2_O_3_-NA and S|NiN_*x*_−PC/EG/Fe_2_O_3_-NA under AM 1.5G irradiation at 1.23 V. All experiments were carried out in 1.0 M NaOH

To further investigate the catalytic mechanism, the population distributions of the related doped graphene materials are presented in Fig. 4a–h. By discussing the overpotential profiles, the structure with the highest catalytic performance is identified as the Ni–N_3_S-doped graphene structure, as seen in Fig. 4g, h. Compared with the Ni–N_4_ model, the S atom in the Ni–N_3_S model is the electron donor^43^ and can reduce the electron donation of the Ni atom to its neighboring N atoms, thus tuning the hybridization states between Ni and the neighboring N atoms, which improves the local electronic structure of the catalytic site and boosts the OER catalytic activity (Fig. 4i–k). Figure 4i shows a typical volcano plot for various active sites on different models in alkaline environments. The values of the calculated OER overpotential are 0.346, 0.461, 0.478, and 0.516 V for the Ni–N_3_S, Ni–N_4_, Ni–S, and N–S models, respectively. The Ni–N_3_S model has the lowest overpotential value. The results indicate that the potential barrier of the third step obviously decreases, and the Ni–N_3_S-doped graphene shows the highest catalytic performance among all models. Owing to the hybridization states with the neighboring C and Ni atoms, the existence of the S atom renders a high positive-charge density and optimizes the density-of-states distributions, which can enhance the electron transfer ability in the Ni–N_3_S model (Fig. 5a)^44^.

The elementary reaction steps towards the OER process over the Ni–N_3_S model in alkaline environments are demonstrated in Fig. 4k. Though the fourth step (OOH* to O_2_ production) is spontaneous, the OER steps have obvious potential barriers from the first to third steps when the electrode potential U is 0 V. When *U* increases to 0.965 V (0.346 V in overpotential), the free energy of elementary reaction steps decrease to below zero, which indicates that the whole OER process can occur spontaneously over this approximate potential. Meanwhile, the catalytic activities of Ni–N_2_S_2_ and Ni–NS_3_ models are studied. Both of them have higher overpotential values than the Ni–N_3_S model. The high potential barriers existing in the transition from O* to OOH* can slow down and even block the O_2_ evolution. The boundary effects of Ni–N_4_ and Ni–N_3_S structures on the armchaired graphene nanoribbon are also investigated. From the calculations, the best catalytic performance for Ni–N_4_ structures is on the center of the armchaired graphene nanoribbon, and the best catalytic performance for Ni–N_3_S structures is on the edge of the armchaired graphene nanoribbon. Thus, the charge redistribution, change in the group adsorption strength, and potential barriers induced by the dopant play important roles in the catalytic activities.

### PEC water oxidation

Based on our DFT calculations (Fig. 5a), the conduction band of Fe_2_O_3_ is close to the Fermi level, and the valence band is slightly lower than the work function of Ni–N_3_S-doped graphene when Ni–N_3_S-doped graphene is integrated into Fe_2_O_3_-NA. This band alignment can promote easier transfer of photogenerated charge carriers between Fe_2_O_3_ and Ni–N_3_S-doped graphene, which facilitates the PEC-OER process (Supplementary Fig. 35)^45,46^. Thus, we further studied S|NiN_*x*_−PC/EG as a co-catalyst with Fe_2_O_3_-NA photoanode (S|NiN_*x*_−PC/EG/Fe_2_O_3_-NA) for solar water oxidation in alkaline solution (AM 1.5G, 100 mA cm^−2^, Supplementary Fig. 36). In Fig. 5b, the S|NiN_*x*_−PC/EG/Fe_2_O_3_-NA delivered a high photocurrent density of 1.58 mA cm^−2^ at 1.23 V, which is 2.59 times larger than the Fe_2_O_3_-NA (0.61 mA cm^−2^), and higher than those reported for other Fe_2_O_3_-based inorganic photoanodes (Supplementary Table 3). Also, a remarkable cathodic shift in the onset potential from 0.8 V for Fe_2_O_3_-NA to 0.7 V for S|NiN_*x*_−PC/EG/Fe_2_O_3_-NA was observed, revealing that the S|NiN_*x*_−PC/EG indeed promoted PEC-OER. The maximum photoconversion efficiency of S|NiN_*x*_−PC/EG/Fe_2_O_3_-NA achieved 0.24% at 0.92 V (Supplementary Fig. 37), tripling that of Fe_2_O_3_-NA (0.07% at 0.96 V). The incident photon-to-current conversion efficiency (IPCE) measurement shows that the S|NiN_*x*_−PC/EG/Fe_2_O_3_-NA possessed a maximum IPCE value of 30.9% at 300 nm at 1.23 V (Fig. 5c), which is about 2.68 times higher than that of Fe_2_O_3_-NA (11.5%).

To understand the effect of S|NiN_*x*_−PC/EG on the promotion of photogenerated charge separation, the charge transport (*η*_transport_) and charge-transfer efficiencies (*η*_transfer_ = *J*_H2O_/*J*_H2O2_) of the S|NiN_*x*_−PC/EG/Fe_2_O_3_-NA were decoupled and quantified by using H_2_O_2_ as a hole scavenger (Supplementary Fig. 38)^47–52^. As shown in Fig. 5d, the addition of S|NiN_*x*_−PC/EG significantly increased the *η*_transfer_ of Fe_2_O_3_-NA throughout the entire potential range. Especially, at 1.23 V, the S|NiN_*x*_−PC/EG/Fe_2_O_3_-NA delivered a much higher *η*_transfer_ of 78.2% than the Fe_2_O_3_-NA (43.6%), illustrating that the S|NiN_*x*_−PC/EG can effectively weaken surface charge recombination and improve charge-transfer from Fe_2_O_3_-NA to electrolyte, thus facilitating water oxidation kinetics^53–56^. The results can be further supported by EIS studies (Supplementary Fig. 39), in which S|NiN_*x*_−PC/EG/Fe_2_O_3_-NA exhibited a much lower charge-transfer resistance than Fe_2_O_3_-NA both in dark and under irradiation, suggesting that more effective interfacial charge-transfer occurred on the S|NiN_*x*_−PC/EG/Fe_2_O_3_-NA interface^57^. Moreover, the S|NiN_*x*_−PC/EG/Fe_2_O_3_-NA exhibited a higher charge transport efficiency (*η*_transport_) of 22.8% at 1.23 V (Fig. 5e), in comparison with the Fe_2_O_3_-NA (*η*_transport_ = 16.3%), which is possibly ascribed to the formed heterojunction between Fe_2_O_3_-NA and S|NiN_*x*_−PC/EG that can facilitate the charge transport in bulk Fe_2_O_3_-NA^49,58^. These results suggest that the introduction of S|NiN_*x*_−PC/EG serving as co-catalyst not only improve bulk charge transport, but it also reduce surface charge recombination, thus increasing the overall efficiency of PEC water oxidation (Supplementary Fig. 40). No significant change in current density of S|NiN_*x*_−PC/EG/Fe_2_O_3_-NA was observed within 10,000 s of irradiation (Fig. 5f), indicating excellent stability.

## Discussion

In summary, we developed a nanocarbon electrocatalyst consisting of an atomic dispersion of a S|NiN_*x*_ complex encapsulated within PC nanosheets. Benefiting from the large surface area, abundant porous architecture, and well-distributed active sites, the resulting S|NiN_*x*_−PC/EG exhibited outstanding OER activity with a low overpotential of 1.51 V at 10 mA cm^−2^ in alkaline media, outperforming all existing transition metal- and/or heteroatom-doped carbon-based electrocatalysts and even surpassing the state-of-the-art Ir/C catalyst. Experimental observations and theoretical calculations reveal that the unusual electrocatalytic activity originates from the optimized density-of-states distribution and enhanced electron transfer ability of the S|NiN_*x*_ active centers confirmed by spherical aberration-corrected electron microscopy and synchrotron radiation X-ray absorption spectroscopy measurements, which synergistically promotes the oxidation kinetics. The integrated S|NiN_*x*_−PC/EG on Fe_2_O_3_-NA photoanode achieved an AM 1.5G photocurrent density of 1.58 mA cm^−2^ at 1.23 V for solar water oxidation, which is higher than those reported for other Fe_2_O_3_-based inorganic photoanodes. Our catalyst design may illuminate the development of novel carbon materials with atomically disperse active molecular entities for diverse PEC applications, including CO_2_ reduction, oxygen reduction, and nitrogen fixation.

## Methods

### Synthesis of S|NiN_*x*_−PC/EG

In a typical experiment, EG was first fabricated by the anodization of graphite foil in 0.1 M (NH_4_)_2_SO_4_ with a Pt counter electrode under 10 V for 15 s^59^. EG was immersed in a 20 mL mixed solution of 0.15 g NiCl_2_•6H_2_O, 0.5 g of dicyanamide, and 0.5 mL thiophene under stirring, and then, they were transferred into a Teflon-lined autoclave for hydrothermal reaction at 200 °C for 4 h. Finally, the obtained TSC/EG electrode was pyrolyzed at 900 °C under a flowing Ar atmosphere for 3 h, followed by acid etching treatment with 0.5 M H_2_SO_4_ to remove unstable nickel species. The loading amount of S|NiN_*x*_−PC/EG on the graphite foil was ~ 0.15 mg cm^−2^. For the comparative study, the control electrodes were also fabricated with similar methods as described above (Supplementary Methods).

### Characterization

Field-emission scanning electron microscope (FESEM) measurements were performed with a Carl Zeiss NVision 40 equipped with an EDX. Transmission electron microscopy (TEM), high-resolution TEM (HRTEM), TEM mapping, and high-angle annular dark-field scanning TEM (HAADF-STEM) were performed on a JEOL JEM-2001F and a Carl Zeiss Libra 120 with double spherical aberration correctors. X-ray diffraction (XRD) patterns were recorded on a Bruker D8 Advance powder diffractometer. X-ray photoelectron spectroscopy (XPS) measurements were performed on an AXIS Ultra DLD system (Kratos). Raman spectra were determined on an NTEGRA spectra system (NT-MDT). N_2_ adsorption–desorption experiments were operated at 77K on a Quadrasorb Adsorption Instrument. Fourier transform infrared spectra (FTIR) were obtained on a BRUKER TENSOR II spectrometer. ^1^H nuclear magnetic resonance (NMR) spectra were recorded with a Bruker DPX 300 spectrometer. Inductively coupled plasma–optical emission spectrometry (ICP-OES) measurements were conducted on a Perkin Elmer Optima 7000DV. The contact angles were measured using a DSA-10 Kruss goniometer. Thermogravimetric analysis (TGA) was performed on a TA SDT 2960 thermoanalyzer. Atomic force microscopy images were taken on an NT-MDT 70 platform (Russia). Ultraviolet (UV)–visible (Vis) spectroscopy was recorded on a Cary 5000 UV–Vis–near infrared (NIR) spectrophotometer. X-ray absorption near-edge structure (XANES) spectra and extended X-ray absorption fine structure (EXAFS) spectra were collected on the BL10C beam line of the Pohang light source (PLS- II) in Korea with a ring current of 100 mA at 3.0 G eV.

### Electrochemical measurements

Electrochemical measurements were performed in a standard three-electrode system (CHI 760E, USA) comprising the S|NiN_*x*_−PC/EG working electrode, a graphite rod counter electrode, and a Ag/AgCl reference electrode. All potentials reported in our work are referenced to a reversible hydrogen electrode (RHE)^60^. The polarization curve was recorded in 1.0 M KOH electrolyte at a scan rate of 1 mV s^−1^. The long-term stability was evaluated by a chronoamperometric measurement. EIS data were collected with frequencies ranging from 100 K to 0.01 Hz under an AC potential of 10 mV.

### PEC measurements

PEC experiments were carried out in a three-electrode configuration in which the S|NiN_*x*_−PC/EG/Fe_2_O_3_-NA with an exposed projected surface area of 1.0 cm^2^ acted as the working electrode. The light source (100 mW cm^−2^) was provided by a 200 W Xenon lamp fitted with an AM 1.5G filter from Newport. The electrolyte used was a 1.0 M NaOH solution. Incident photon-to-current conversion efficiencies (IPCE) were measured by using monochromatic irradiation from the Xe lamp equipped with an aligned monochromator.

### Computational details

All DFT calculations were conducted by using the Cambridge Serial Total Energy Package (CASTEP) in Material Studio, which is based on the DFT plane-wave pseudopotential approach. The computational details are in the Supporting Information.

## Supplementary information

Supplementary Information

## Data Availability

The data that support the findings of this study are available from the corresponding author upon reasonable request.
