# Supplementary material for: Atomically dispersed nickel–nitrogen–sulfur species anchored on porous carbon nanosheets for efficient water oxidation
Source: Nat Commun. 2019 Mar 27;10:1392. doi: 10.1038/s41467-019-09394-5 (PMC6437202; doi:10.1038/s41467-019-09394-5)
Supplement: Supplementary file 1 — Supplementary Information [file 41467_2019_9394_MOESM1_ESM.pdf]

Supplementary Information

Atomically Dispersed Nickel–Nitrogen–Sulfur Species Anchored on Porous Carbon Nanosheets for Efficient Water Oxidation

Yang Hou et al

Supplementary Figures

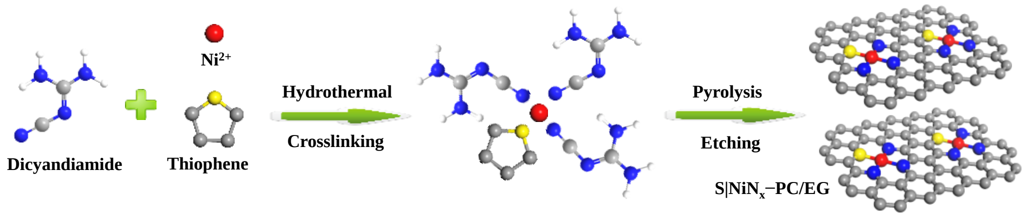

Supplementary Figure 1. **Design and synthesis of the S|NiN$_x$−PC/EG.** Schematic illustration for the synthesis process of S|NiN$_x$−PC/EG.

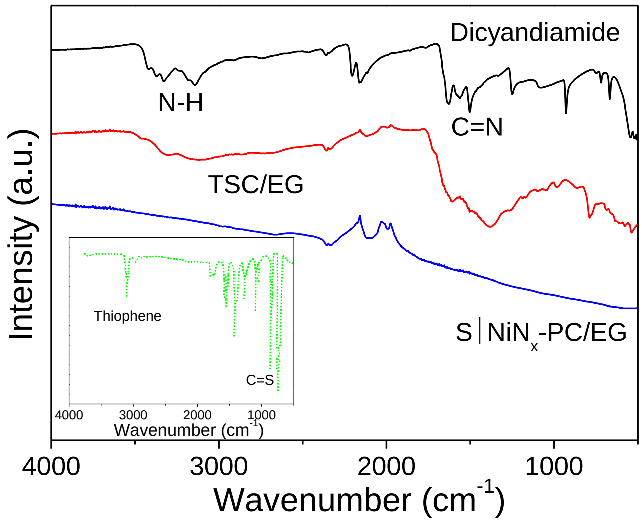

Supplementary Figure 2. **FTIR characterization.** FTIR spectra of dicyandiamide, thiophene, TSC/EG, and S|NiN_x–PC/EG. Data for thiophene is from NIST Chemistry WebBook, SRD 69 (http://webbook.nist.gov/cgi/cbook.cgi?ID=C110021&Mask=80).

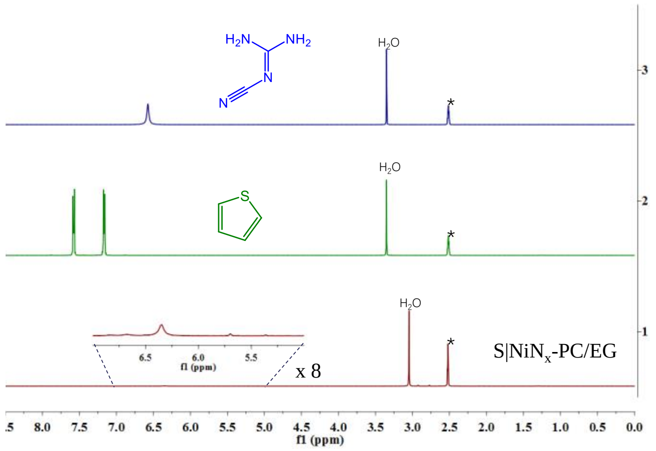

Supplementary Figure 3. **NMR characterization.** ^{1}H-NMR spectra (300 MHz, DMSO-d6) of dicyandiamide (blue curve), thiophene (green curve), and S|NiN$_x$−PC/EG (brown curve).

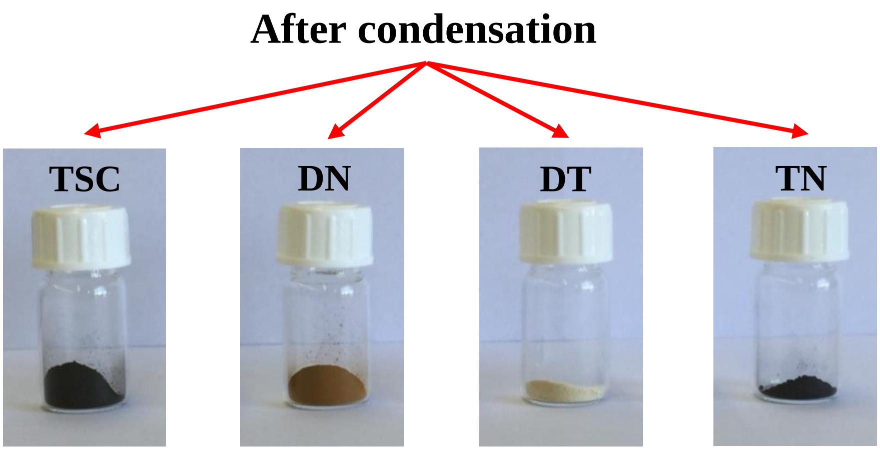

Supplementary Figure 4. **Color comparison between four samples**. Digital photos of powder TSC, powder DN, powder DT, and powder TN.

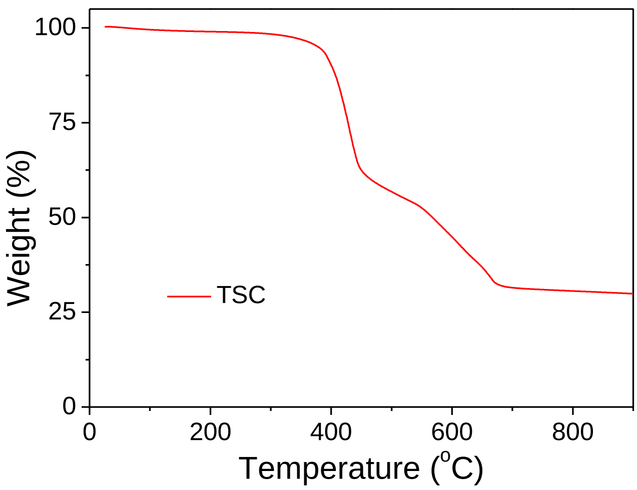

Supplementary Figure 5. **TGA characterization.** TGA curve of TSC under flowing Ar.

The first two stages of weight loss occurred at 100-390 °C and 390-450 °C. The corresponding weight loss can be ascribed to the removal of adsorbed water and partial carbonization of the TSC.[1] The third weight loss of ~ 30.8% in the range of 450-690 °C was caused by structure decomposition of TSC to form S|NiN_x−PC with the incorporation of Ni nanoparticles. During the pyrolysis process, Ni species are reduced in situ by carbonization of the organic linker, and the N- and S-containing frameworks in the TSC lead to simultaneous co-doping of N and S into the resultant carbon matrix, leading to the formation Ni atoms anchored on N and S species.

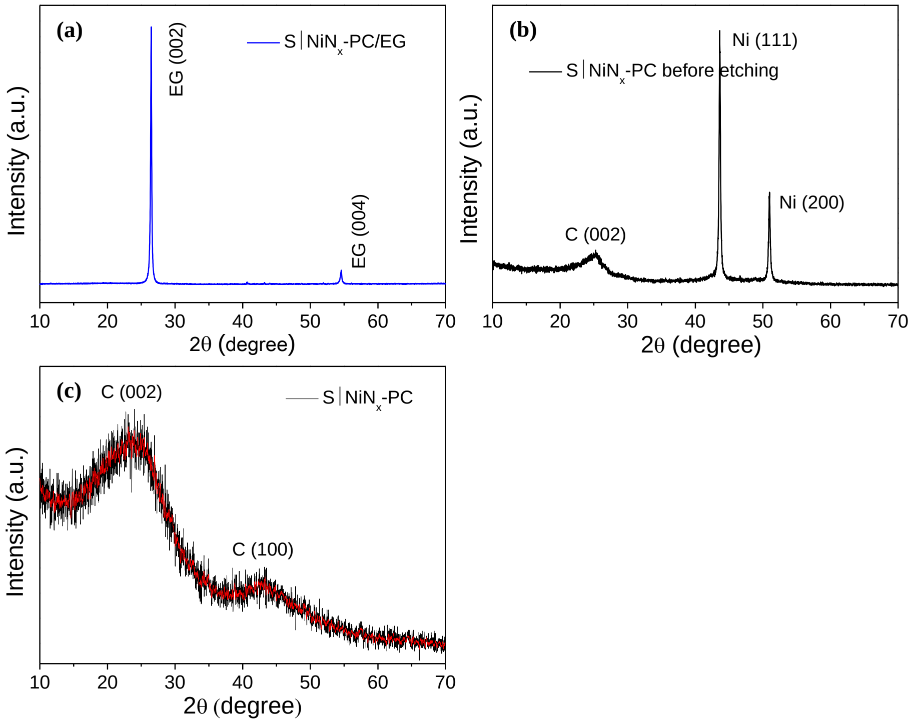

Supplementary Figure 6. **XRD characterization.** XRD patterns of S|NiN_x−PC/EG (a), S|NiN_x−PC before etching (b), and S|NiN_x−PC (c).

XRD analyses of S|NiN_x−PC/EG showed two distinct characteristic peaks at 24.5 and 43.8^o, originating from the (002) and (100) planes of graphitic carbon. For the S|NiN_x−PC before etching, the peak at about 25.2 ° corresponds to the (002) plane of graphitic carbon, while the other peaks at around 43.6 and 51.0 ° are attributed to metallic Ni (111) and Ni (200),[2] respectively, which indicates the presence of metallic Ni in the S|NiN_x−PC prepared without acid leaching treatment.

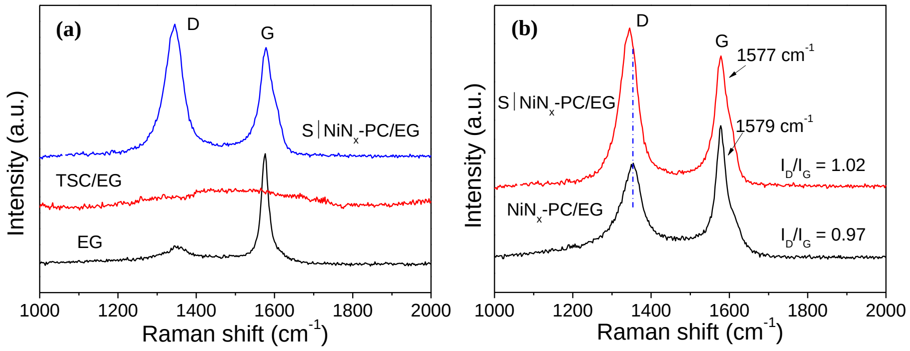

Supplementary Figure 7. Raman characterization. Raman spectra of S|NiN_x−PC/EG (a), TSC/EG (a), EG (a), and NiN_x−PC/EG (b).

As shown in Figure 1a and Supplementary Figure 7, the well-resolved D (1,344 cm^{-1}), G (1,577 cm^{-1}), and 2D (2,689 cm^{-1}) bands with an I_D/I_G ratio of 1.02 in Raman spectrum corroborated the graphitic nature of S|NiN_x−PC/EG. With the introduction of S species, the G band of S|NiN_x−PC/EG shifts toward a lower wavenumber of 1,577 cm^{-1} together with an increase in the I_D/I_G ratio (1.02), in comparison to that of NiN_x−PC/EG (0.97), indicating that the S incorporation can induce the formation of more defective structures in the S|NiN_x−PC/EG,[3] which is helpful for providing enhanced electrocatalytic activities.

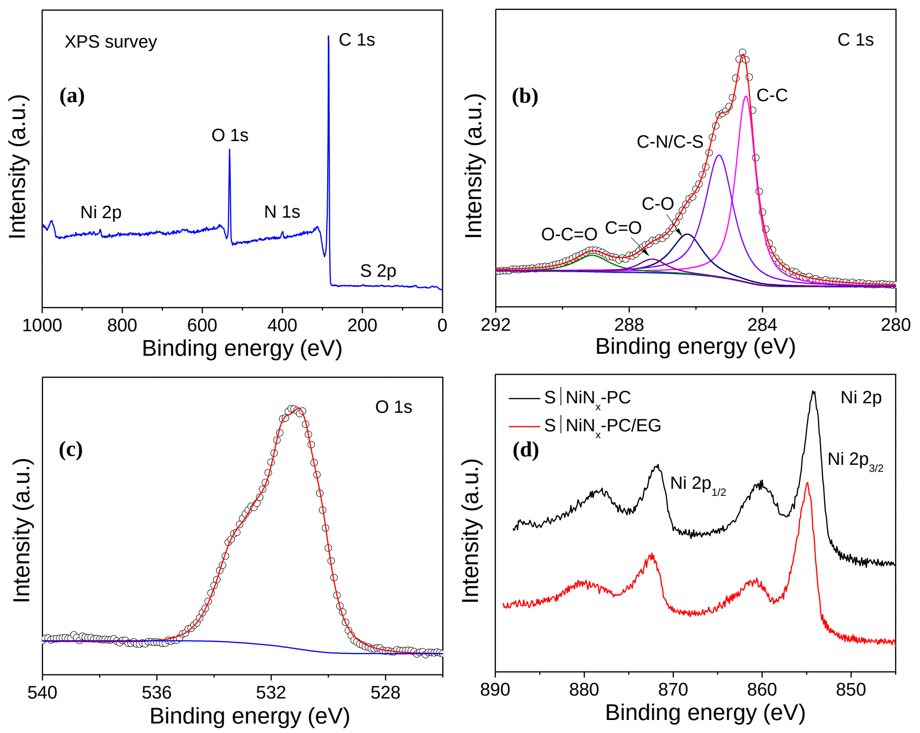

Supplementary Figure 8. **XPS characterization.** The XPS survey spectrum (a), high-resolution C 1s XPS spectrum (b), and high-resolution O 1s XPS spectrum (c) of S|NiN_x−PC/EG. (d) The high-resolution Ni 2p XPS spectra of S|NiN_x−PC and S|NiN_x−PC/EG.

The high-resolution C 1s spectrum of S|NiN_x−PC/EG can be deconvoluted into five peaks at 284.5, 285.3, 286.3, 287.2, and 289.1 eV, corresponding to the C–C, C–N/C–S, C–O, C=O, and C–O=C bonds (Supplementary Figure 8b), respectively. The existence of C–N/C–S bonds supports the N/S-doping in the S|NiN_x−PC/EG. The high-resolution Ni 2p spectra of S|NiN_x−PC/EG slightly shift to higher binding energy range compared with that of S|NiN_x−PC (Supplementary Figure 8d), implying the strong electronic interactions between S|NiN_x−PC and EG in the hybrid.

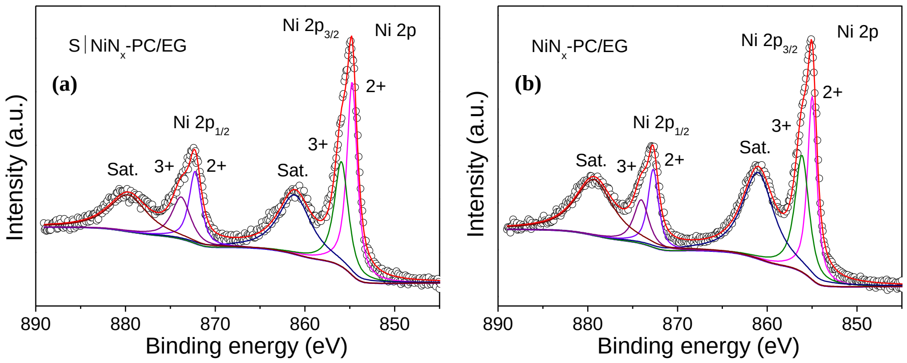

Supplementary Figure 9. **XPS characterization.** The high-resolution Ni 2p XPS spectra of S|NiN_x–PC/EG (a) and NiN_x–PC/EG (b).

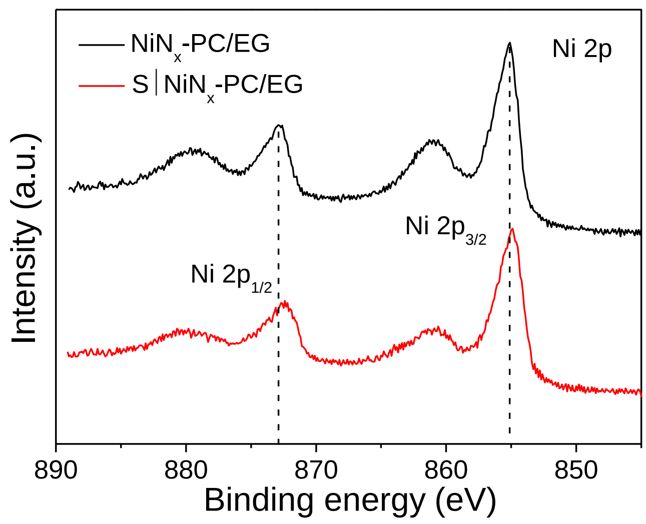

Supplementary Figure 10. **XPS characterization.** The high-resolution Ni 2p XPS spectra of NiN_x–PC/EG and S|NiN_x–PC/EG.

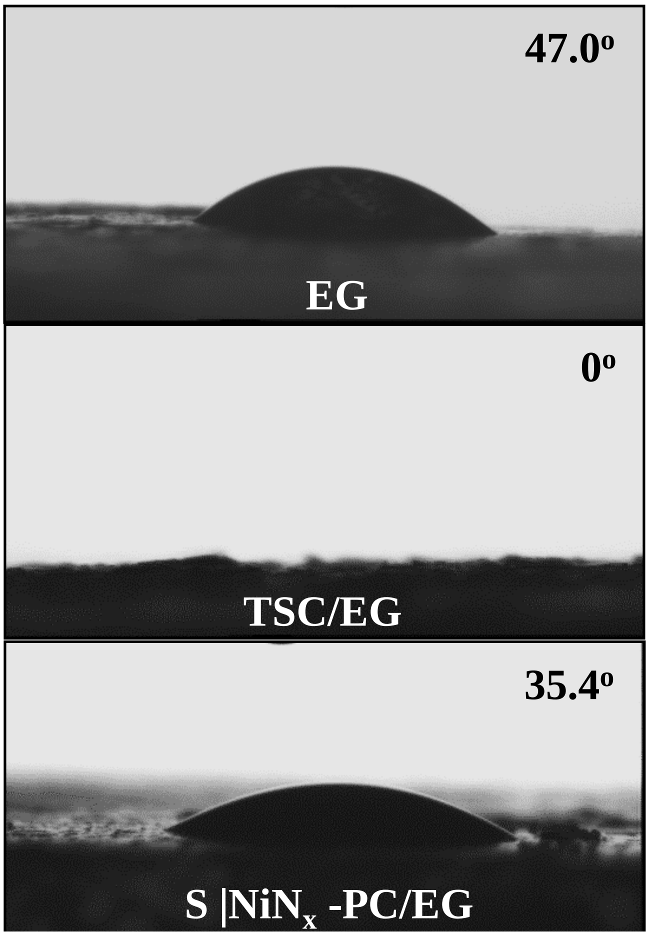

Supplementary Figure 11. **Contact wetting angels characterization.** Contact wetting angels of EG, TSC/EG, and S|NiN$_x$−PC/EG.

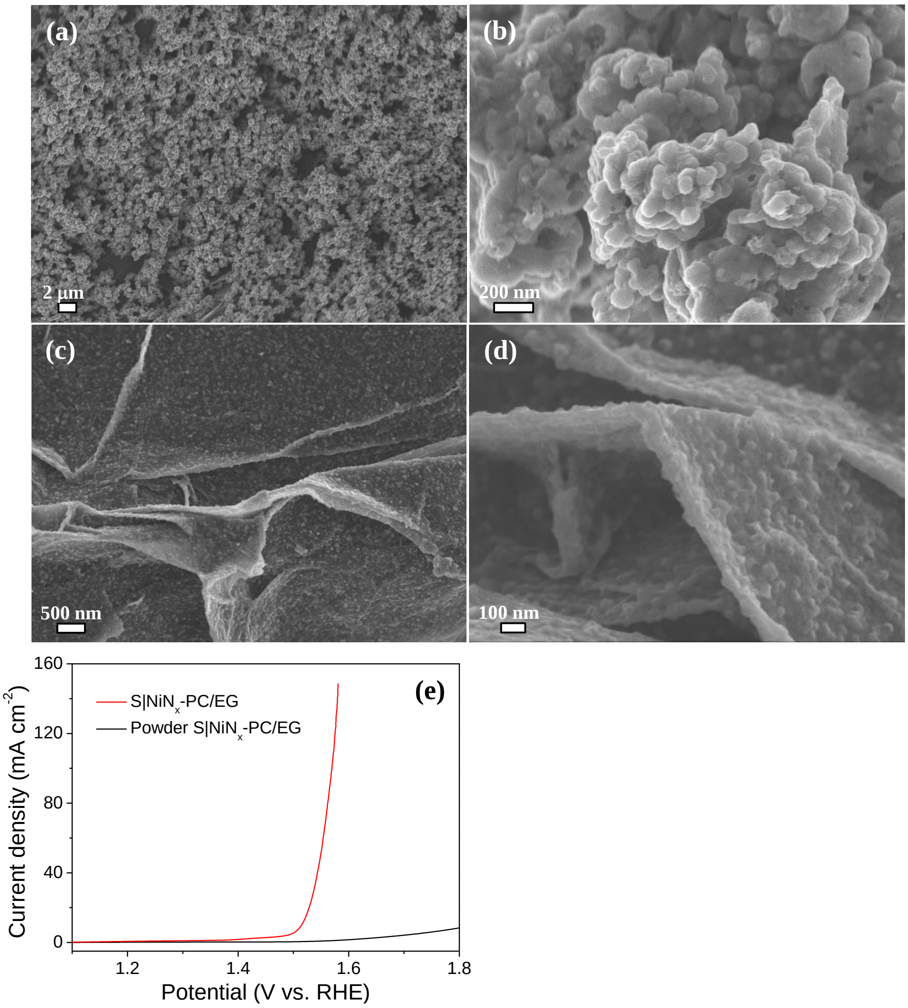

Supplementary Figure 12. FESEM characterization and activity comparison between two electrodes. FESEM images of S|NiN$_x$−PC (a-b) and S|NiN$_x$−PC/EG (c-d). (e) Polarization curves of the S|NiN$_x$−PC/EG and powder S|NiN$_x$−PC/EG obtained by scratching down the S|NiN$_x$−PC/EG from graphite foil and then deposited on glassy carbon electrode in 1.0 M KOH.

Under the same conditions, only irregular S|NiN$_x$−PC nanoparticles were obtained when the EG supports were not used. These intrinsic advantages make the integrated S|NiN$_x$−PC/EG more attractive than its powder counterpart for OER (Supplementary Figure 12).

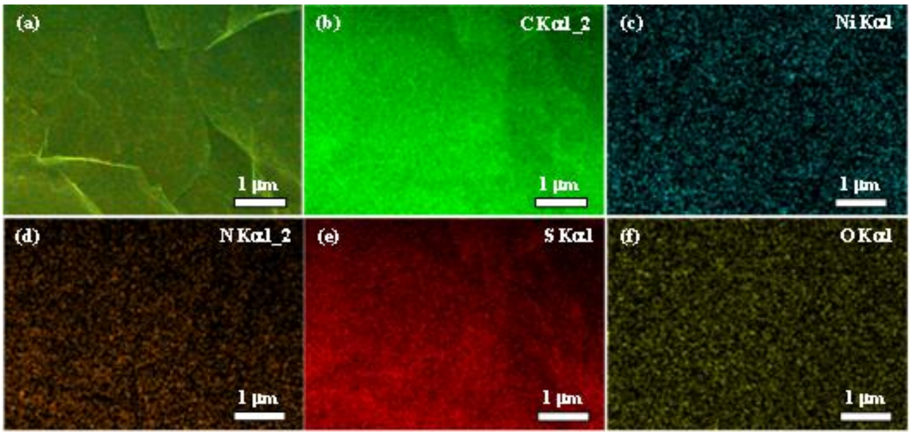

Supplementary Figure 13. **FESEM and EDX mappings characterization.** FESEM image (a) and corresponding EDX mappings (b-f) of S|NiN_x−PC/EG.

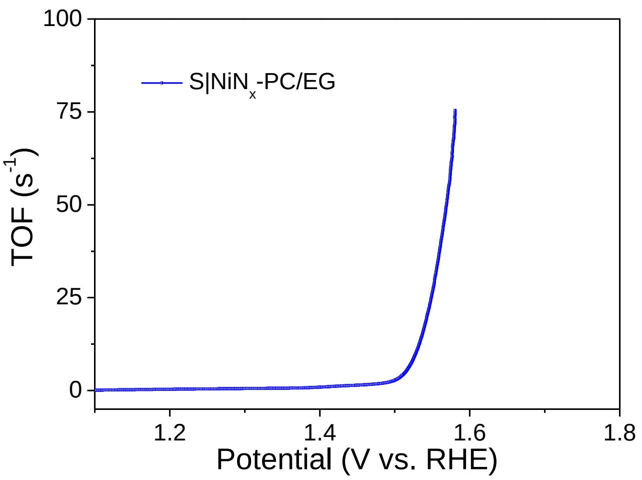

Supplementary Figure 14. **TOF result.** The calculated TOF for S|NiN$_x$−PC/EG. Assuming all the Ni sites were electrochemically active in the OER process, the TOF value of S|NiN$_x$−PC/EG was calculated to be 10.9 s^{-1} at 1.53 V, which was much higher than the TOF values of the other reported non-noble metal OER electrocatalysts.

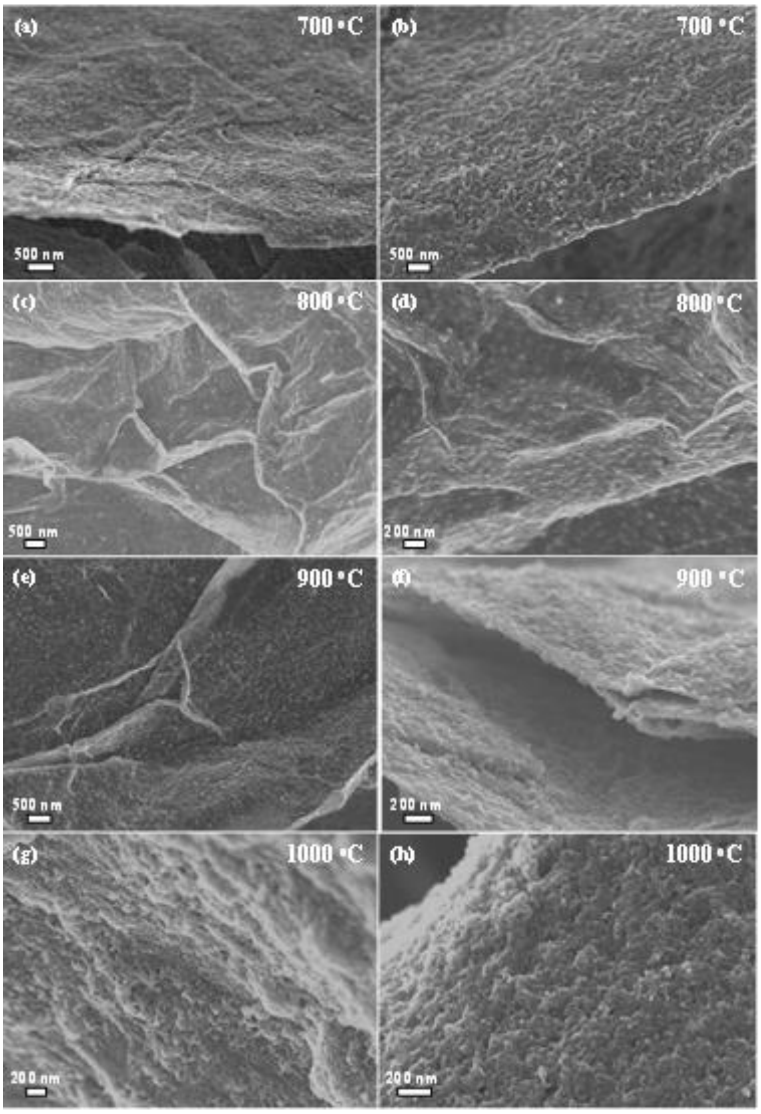

Supplementary Figure 15. **FESEM characterization.** FESEM images of S|NiN$_x$−PC/EG at different carbonization temperature. (a-b) S|NiN$_x$−PC/EG (700 ^{o}C), (c-d) S|NiN$_x$−PC/EG (800 ^{o}C), (e-f) S|NiN$_x$−PC/EG (900 ^{o}C), and (g-h) S|NiN$_x$−PC/EG (1,000 ^{o}C).

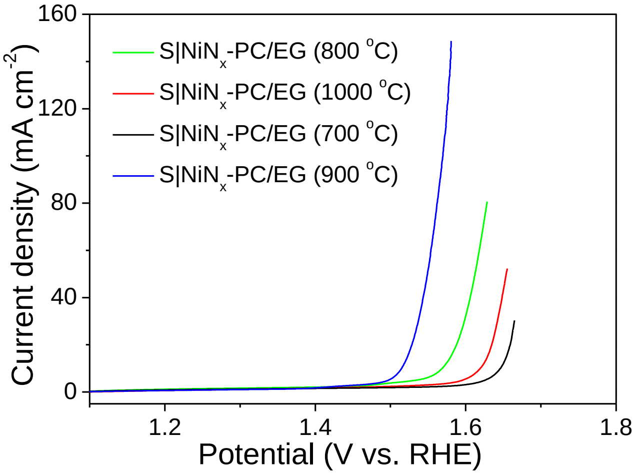

Supplementary Figure 16. **Activity comparison of the different S|NiN$_x$−PC/EG electrodes.** Polarization curves of S|NiN$_x$−PC/EG (700 ^{o}C), S|NiN$_x$−PC/EG (800 ^{o}C), S|NiN$_x$−PC/EG (900 ^{o}C), and S|NiN$_x$−PC/EG (1,000 ^{o}C) in 1.0 M KOH.

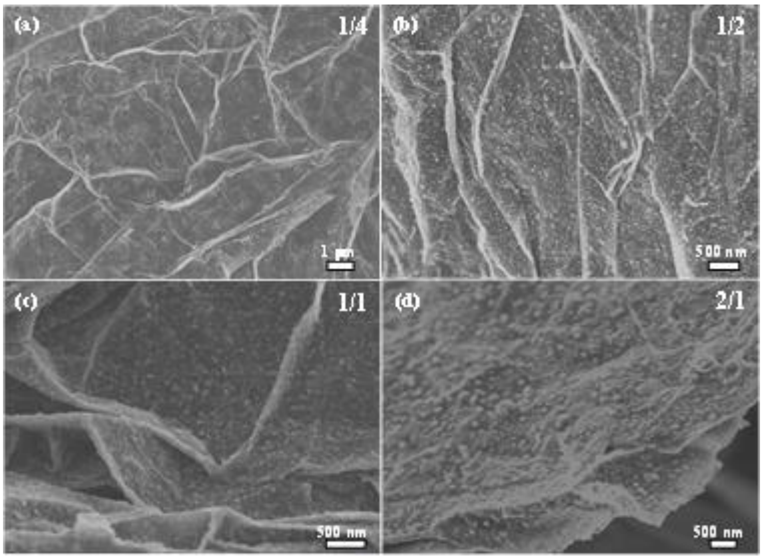

Supplementary Figure 17. **FESEM characterization.** FESEM images of S|NiN$_x$−PC/EG with varying amounts of Ni-precursors. (a) S|NiN$_x$−PC/EG-1/4Ni, (b) S|NiN$_x$−PC/EG-1/2Ni, (c) S|NiN$_x$−PC/EG, and (d) S|NiN$_x$−PC/EG-2/1Ni.

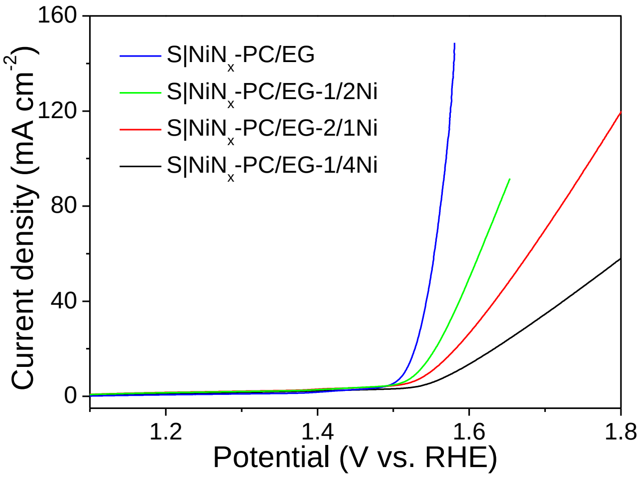

Supplementary Figure 18. **Activity comparison of the different S|NiN$_x$−PC/EG electrodes.** Polarization curves of S|NiN$_x$−PC/EG, S|NiN$_x$−PC/EG-1/4Ni, S|NiN$_x$−PC/EG-1/2Ni, and S|NiN$_x$−PC/EG-2/1Ni in 1.0 M KOH.

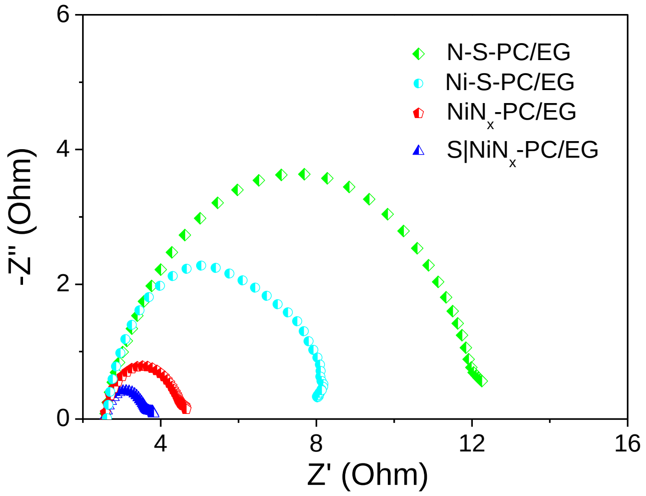

Supplementary Figure 19. **EIS activity comparison between four electrodes.** EIS Nyquist plots of N-S-PC/EG, Ni-S-PC/EG, NiN_x–PC/EG, and S|NiN_x–PC/EG.

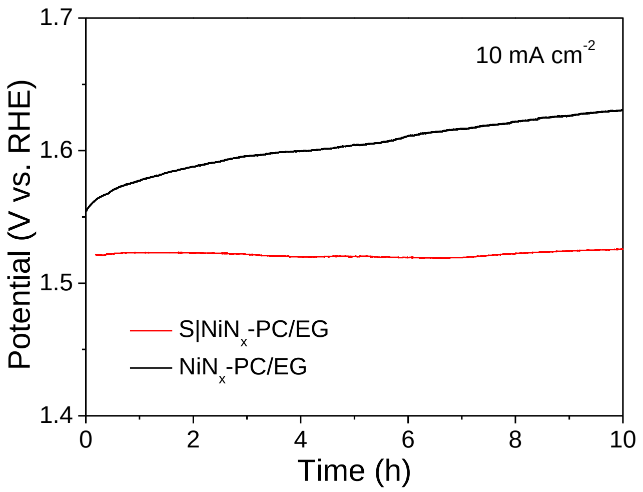

Supplementary Figure 20. **Stability comparison between two electrodes.** Chronopotentiometry curves of NiN_x–PC/EG and S|NiN_x–PC/EG under a current density of 10 mA cm^{-2} in 1.0 M KOH. In contrast, the NiN_x–PC/EG was less stable under the same condition, indicating that the incorporation of S atom greatly enhances the whole catalytic durability.

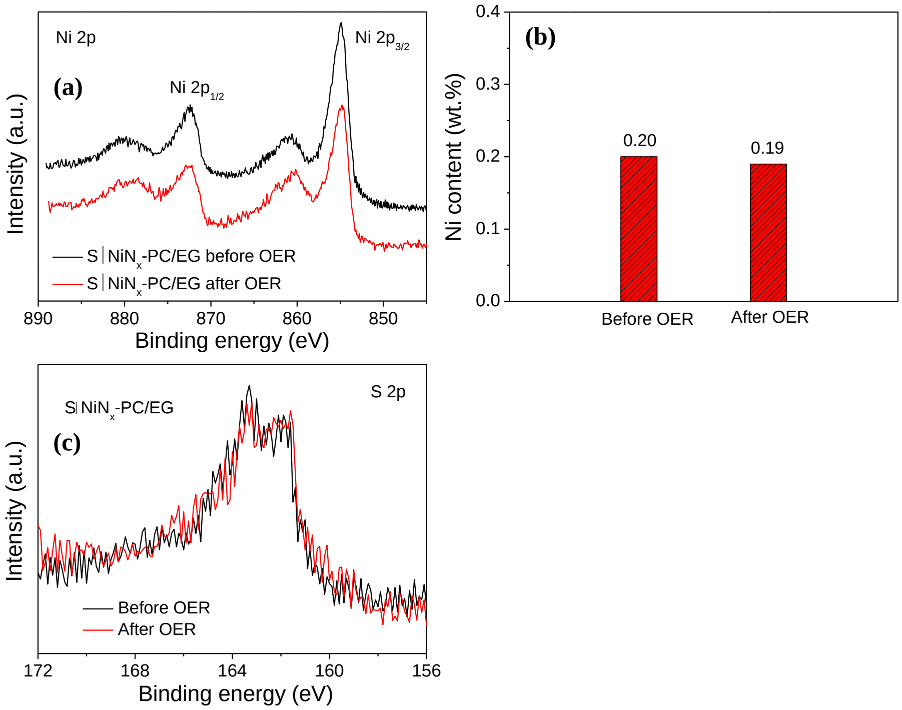

Supplementary Figure 21. XPS characterization. (a) The high-resolution Ni 2p XPS spectra of S|NiN$_x$−PC/EG before and after OER, (b) Ni-content column bar graph of S|NiN$_x$−PC/EG before and after OER, and (c) the high-resolution S 2p XPS spectra of S|NiN$_x$−PC/EG before and after OER.

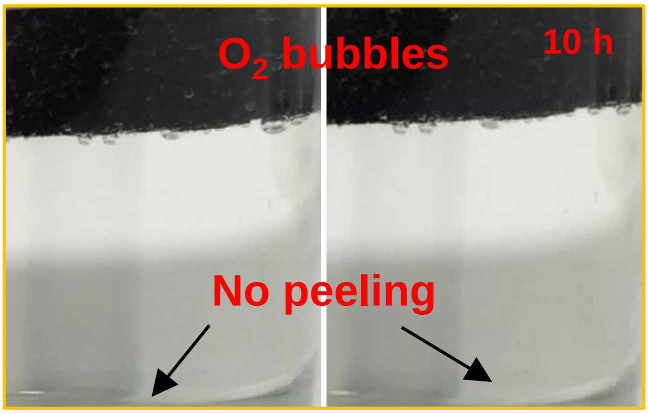

Supplementary Figure 22. Stability characterization. The optical images of S|NiN_x−PC/EG electrode during OER reaction. For the stability test, even on operation at a current density of 10 mA cm^{-2}, the S|NiN_x−PC/EG shows no visible peeling from graphite foil during the evolution of a large amount of O_2 gas, suggesting the strong stability of the S|NiN_x−PC/EG electrode, which is a highly desired property for the practical application of OER catalysts.[4]

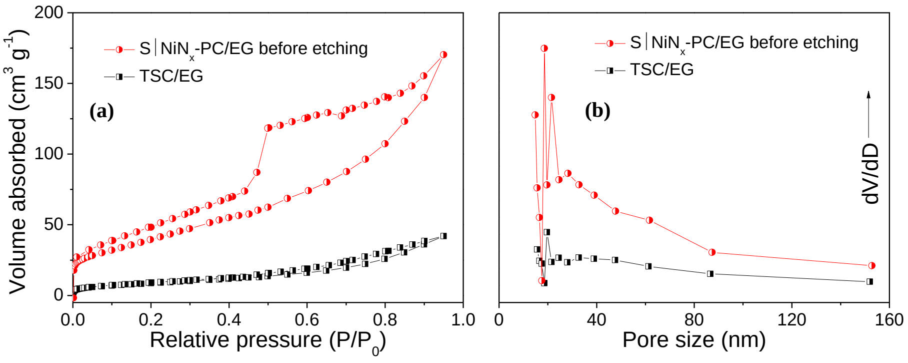

Supplementary Figure 23. **BET characterization.** N_2 adsorption isotherms (a) and the corresponding pore size distributions (b) of TSC/EG and S|NiN_x−PC/EG before etching.

It was found that the S|NiN_x−PC/EG shows a relatively high BET surface area of 235 m^2 g^{-1} compared with the S|NiN_x−PC/EG before etching (134 m^2 g^{-1}), indicating that the acid leaching treatment efficiently removes Ni nanoparticles, which resulted in an increased BET surface area. The high BET surface area for the S|NiN_x−PC/EG is anticipated to provide more active sites, thus facilitating the high electrocatalytic activity.

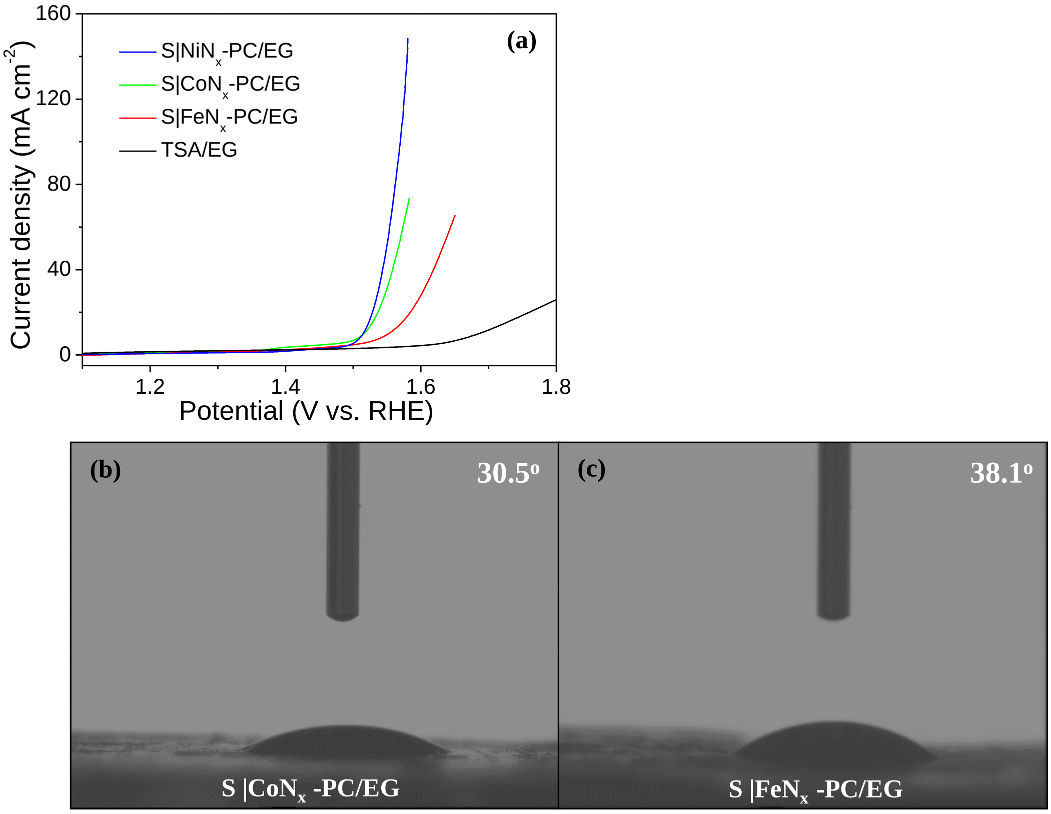

Supplementary Figure 24. Activity comparison between four electrodes and contact wetting angels characterization. (a) Polarization curves of S|NiN$_x$−PC/EG, S|CoN$_x$−PC/EG, S|FeN$_x$−PC/EG, and TSC/EG in 1.0 M KOH. (b-c) Contact wetting angels of S|CoN$_x$−PC/EG and S|FeN$_x$−PC/EG.

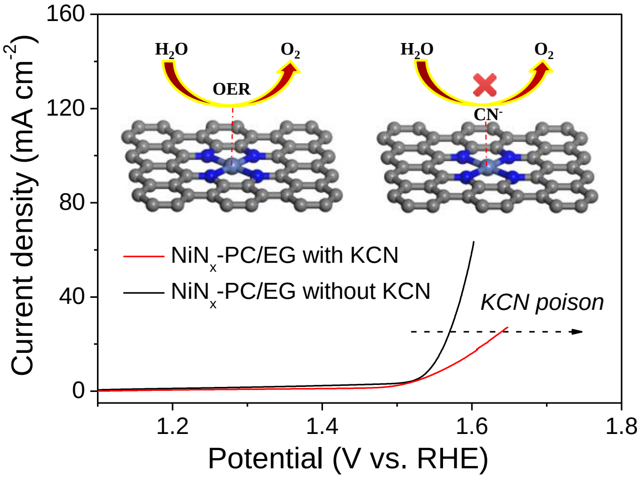

Supplementary Figure 25. **Understanding the structure of active sites.** Polarization curves of NiN_x–PC/EG with and without 10 mM KCN in 1.0 M KOH. Inset: illustrations of NiN_x centers blocked by the CN^{-} ions.

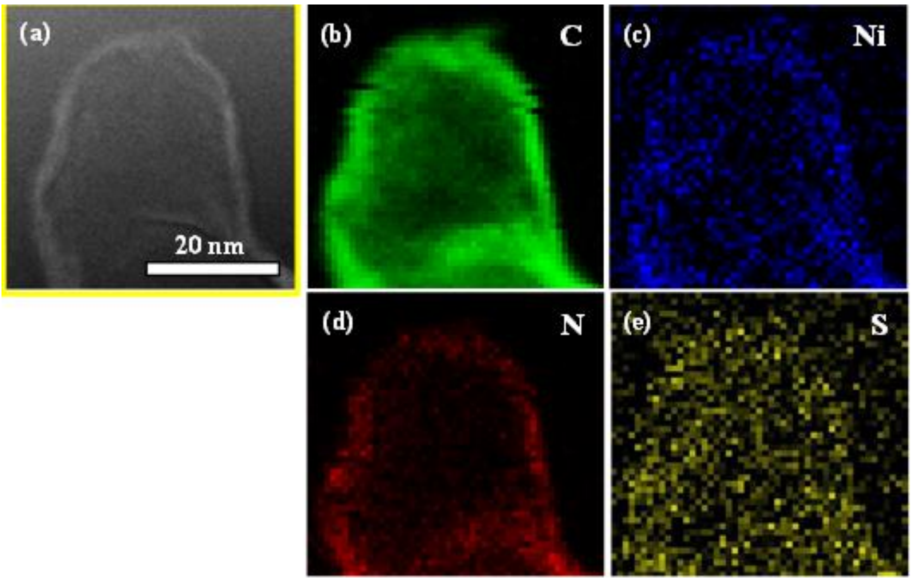

Supplementary Figure 26. **HAADF-STEM and EDX mappings characterization.**

(a) HAADF-STEM image and (b-e) corresponding EDX mappings of S|NiN_x−PC/EG.

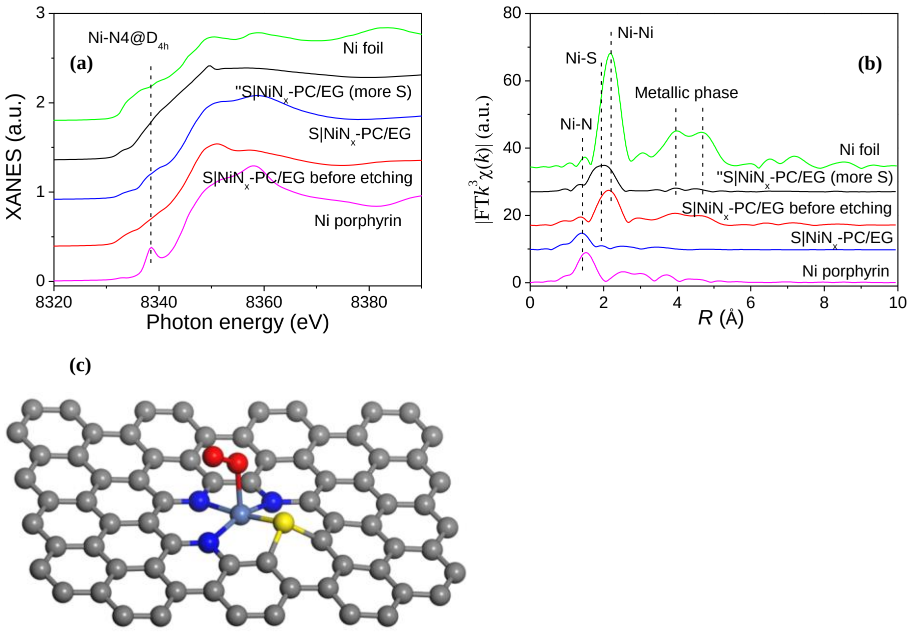

Supplementary Figure 27. **XANES and EXAFS characterization.** (a) XANES spectra and (b) Fourier transform (FT) of the Ni K-edge and wavelet transform (WT) of S|NiN$_x$−PC/EG, S|NiN$_x$−PC/EG before etching, ”S|NiN$_x$−PC/EG (more S), Ni porphyrin, and Ni foil. (c) Schematic model of S|NiN$_x$−PC/EG, Ni (steel blue), N (blue), S (yellow), C (gray), and O (red).

On the other hand, the RDF of S|NiN$_x$−PC/EG before etching shows a partial phase transition to Ni metallic phase, although there is an existence of Ni-N/S chemical bonding from XANES feature similar to S|NiN$_x$−PC/EG. In comparison to RDFs, the existence of Ni metallic phase is relatively dominant in S|NiN$_x$−PC/EG before etching rather than S|NiN$_x$−PC/EG, indicating that the leaching process is efficient to remove the metallic Ni species.

In comparison to RDFs, the Ni-S chemical bonding character remain more in ”S|NiN$_x$−PC/EG (more S) than that of S|NiN$_x$−PC/EG, confirming that the amount of S species in the ”SNiN$_x$−PC/EG (more S) was much higher than that for the S|NiN$_x$−PC/EG catalyst. In other words, the existence of more S-rich element in ”S|NiN$_x$−PC/EG (more S) than Ni−N$_3$S composition in S|NiN$_x$−PC/EG results in the abrupt increase of FT peak of S element. Notably, the small excess amount of S species led to formation of metallic Ni phase, which changes the atomic structure of the S|NiN$_x$ centers in the carbon matrix.

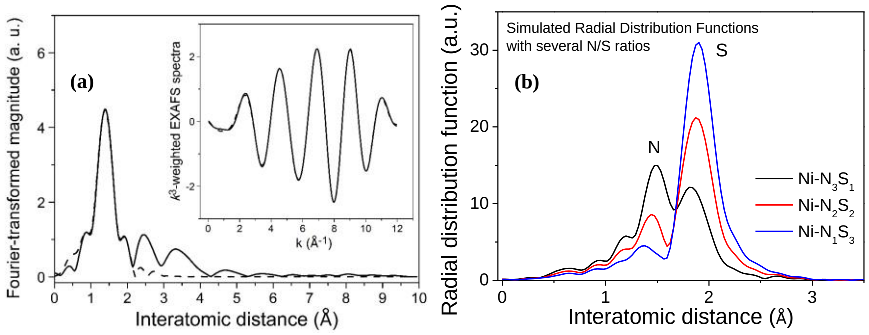

Supplementary Figure 28. **EXAFS characterization.** (a) Experimental (solid line) and fitted (dashed line) radial distribution functions for the S|NiN$_x$−PC/EG. Inset figure shows corresponding to Fourier-filtered (solid line) and fitted (dashed line) k^3-weighted EXAFS spectra. (b) Simulated radial distribution functions with Ni–N_3S_1, Ni–N_2S_2, and Ni–N_1S_3 models.

In Supplementary Figure 28b, the existence of more S-rich element than Ni-N_3S_1 composition presents an abrupt increase of FT peak of S element. The intensive FT feature is originated from the higher backscattering amplitude by the heavier electron density of sulfur atom than nitrogen. When the mole ratio of sulfur increases from one to three, the FT peak intensity of S-element increases beyond the chemical composition (three times), and the FT peak intensity of N-element decreases much severely. Instead of Ni-N_2S_2 or Ni-NS_3 models, it is expected that no reacted Ni-N_4 porphyrin element with S can remain as a minor composition with major Ni-N_3S_1 model. As we know, the absolutely-correct chemical composition of Ni-N_nS_m in the synthetic electrocatalyst might be only one. However, the atomic-selective spectroscopic approach could figure out averaged major chemical composition of Ni-N_3S_1 model in the S-substituted Ni-N_nS_m in the synthetic electrocatalyst.

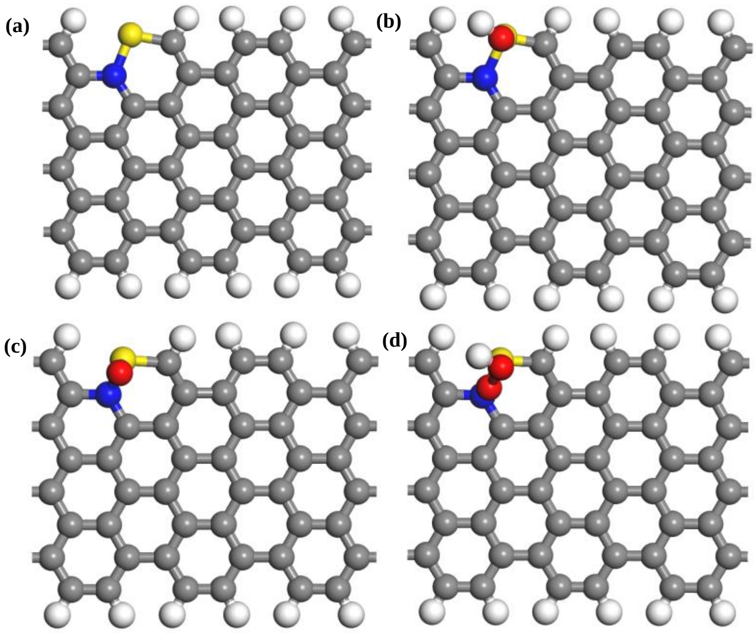

Supplementary Figure 29. **DFT calculation results.** Optimized chemical structures before (a) and after the adsorption of OH^- (b), O^- (c), and OOH^- (d) on N-S doped armchaired nanoribbon.

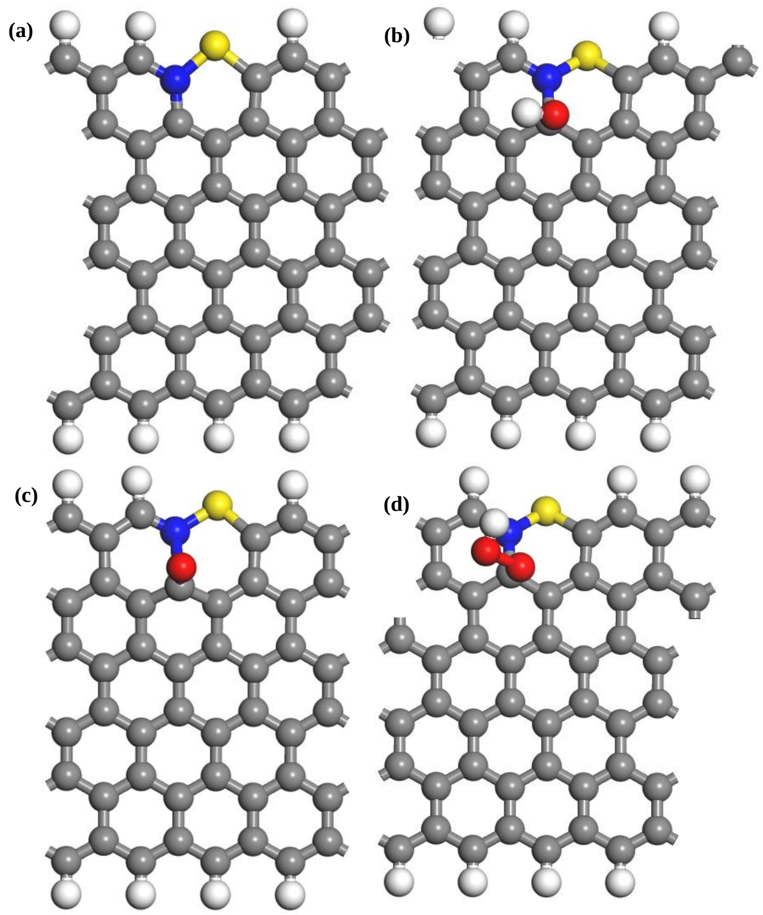

Supplementary Figure 30. **DFT calculation results.** Optimized chemical structures before (a) and after the adsorption of OH^- (b), O^- (c), and OOH^- (d) on N-S doped zigzag nanoribbon.

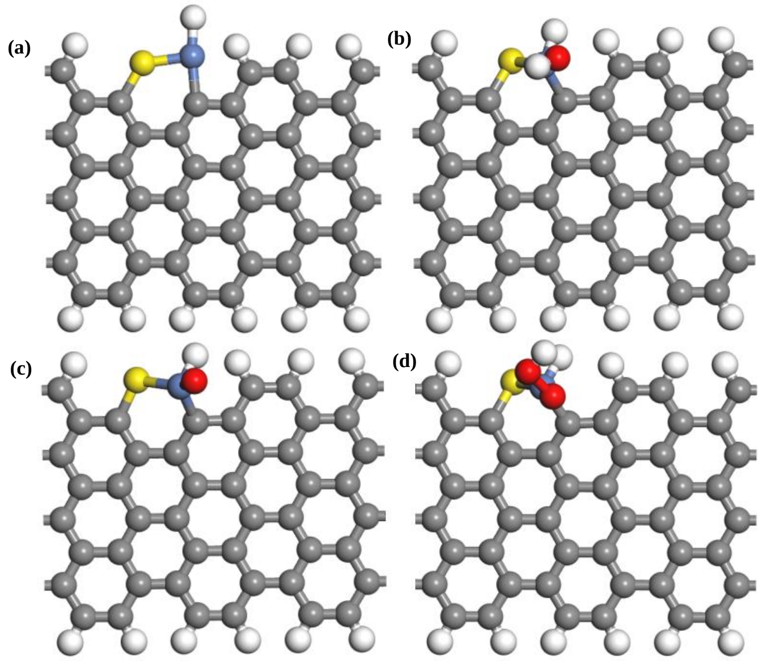

Supplementary Figure 31. **DFT calculation results.** Optimized chemical structures before (a) and after the adsorption of OH^- (b), O^- (c), and OOH^- (d) on Ni-S doped armchaired nanoribbon.

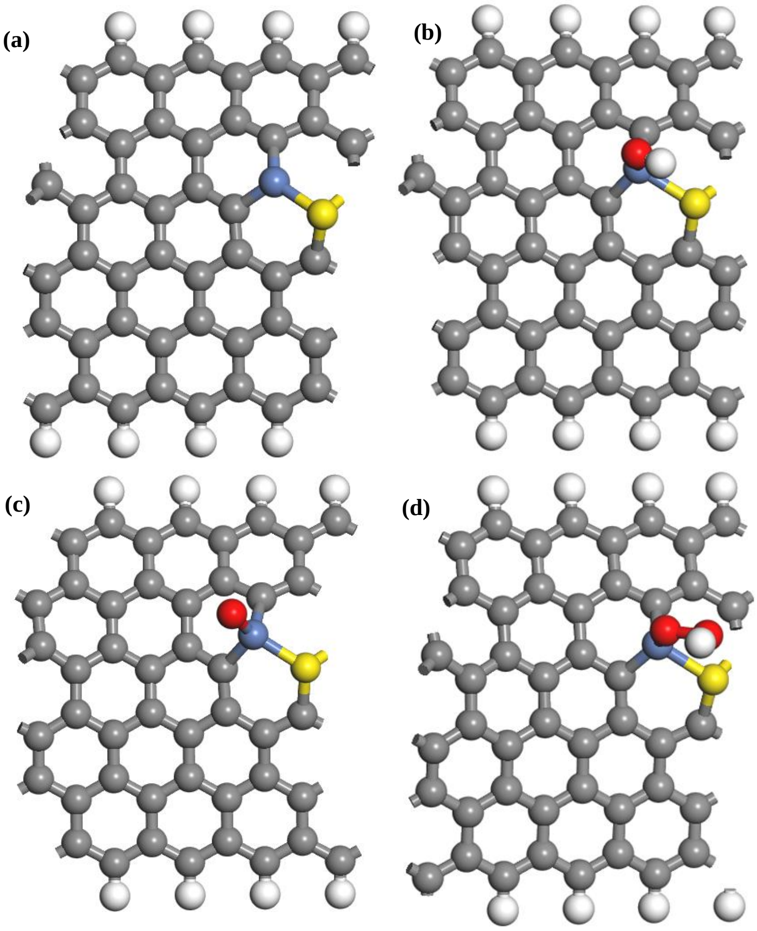

Supplementary Figure 32. **DFT calculation results.** Optimized chemical structures before (a) and after the adsorption of OH^- (b), O^- (c), and OOH^- (d) on Ni-S doped zigzag nanoribbon.

(a) (b) (c) (d)

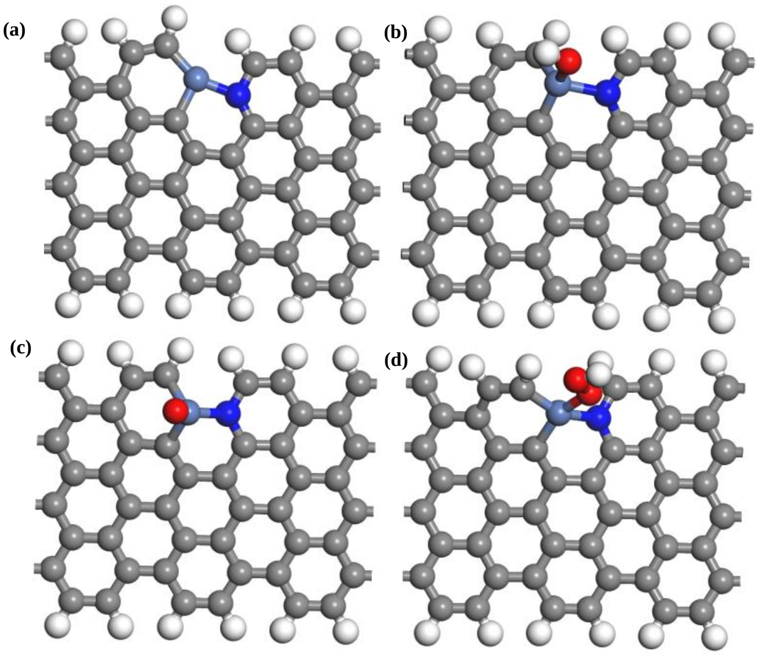

Supplementary Figure 33. **DFT calculation results.** Optimized chemical structures before (a) and after the adsorption of OH^- (b), O^- (c), and OOH^- (d) on Ni-N doped armchaired nanoribbon.

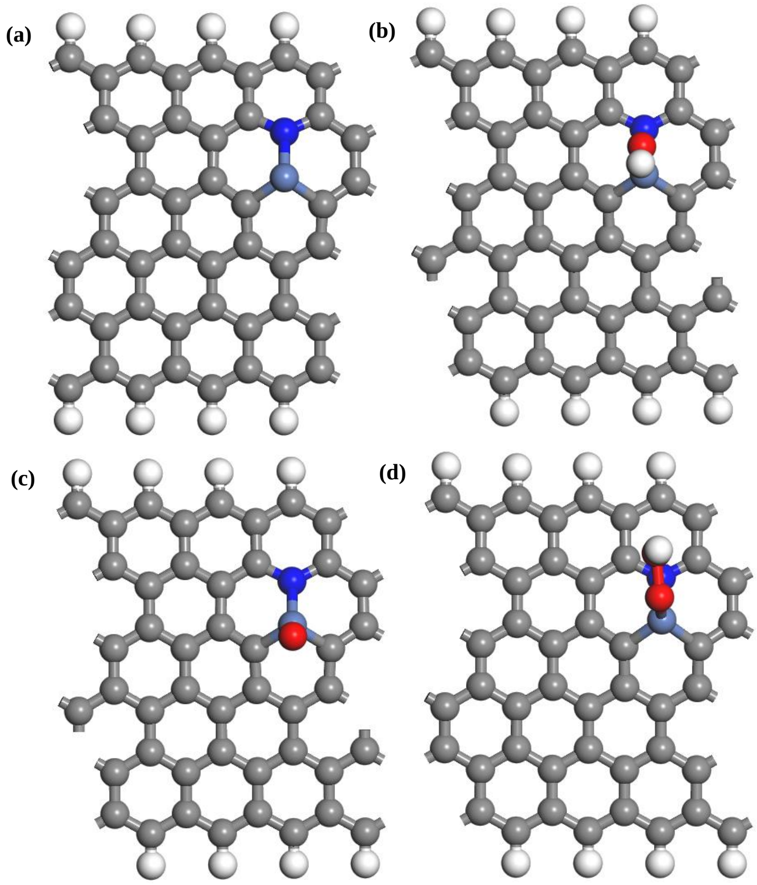

Supplementary Figure 34. **DFT calculation results.** Optimized chemical structures before (a) and after the adsorption of OH^- (b), O^- (c), and OOH^- (d) on Ni-N doped zigzag nanoribbon.

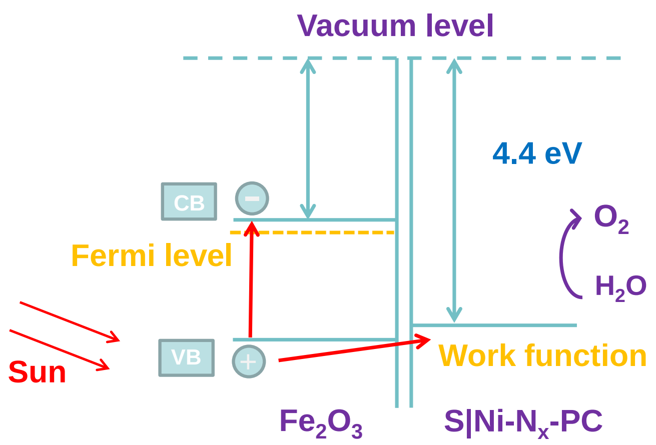

Supplementary Figure 35. PEC-OER mechanism. Schematic illustration for PEC-OER process in the S|NiN$_x$−PC/EG/Fe_2O_3-NA.

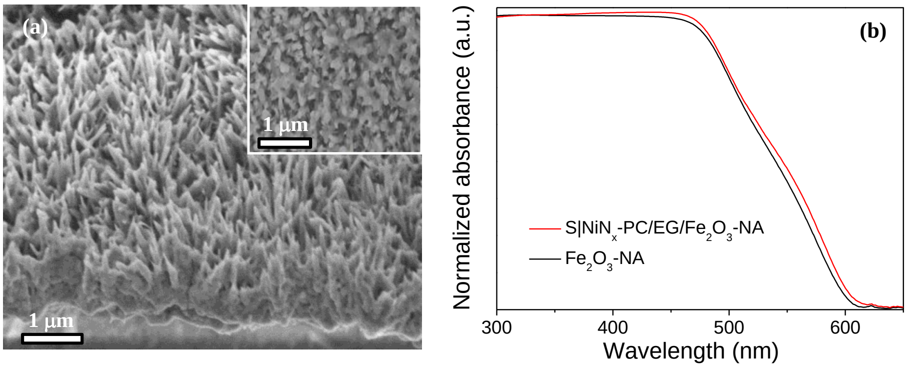

Supplementary Figure 36. **FESEM characterization and light absorption characterization.** (a) Side-viewed and top-viewed (inset) FESEM images of S|NiN_x−PC/EG/Fe_2O_3-NA. (b) Normalized light absorption spectra of S|NiN_x−PC/EG/Fe_2O_3-NA and Fe_2O_3-NA.

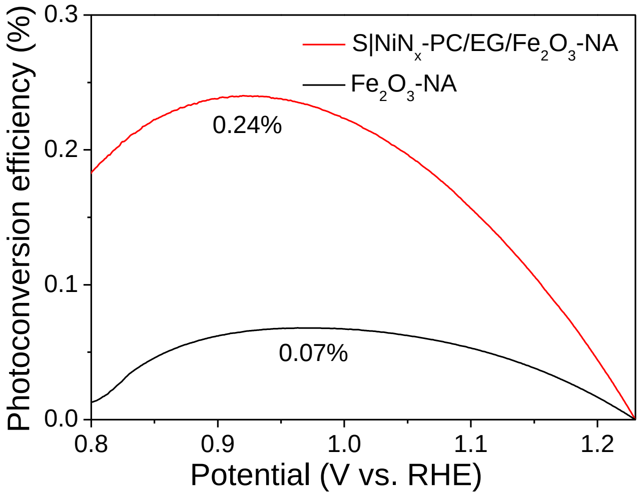

Supplementary Figure 37. **Photoconversion efficiency comparison between two electrodes.** Photoconversion efficiency as a function of applied potential for S|NiN_x–PC/EG/Fe_2O_3-NA and Fe_2O_3-NA under AM 1.5G irradiation in 1.0 M NaOH. The corresponding photoconversion efficiency of S|NiN_x–PC/EG/Fe_2O_3-NA was 0.24% at 0.92 V, which is much greater than that of Fe_2O_3-NA (0.07% at 0.96 V).

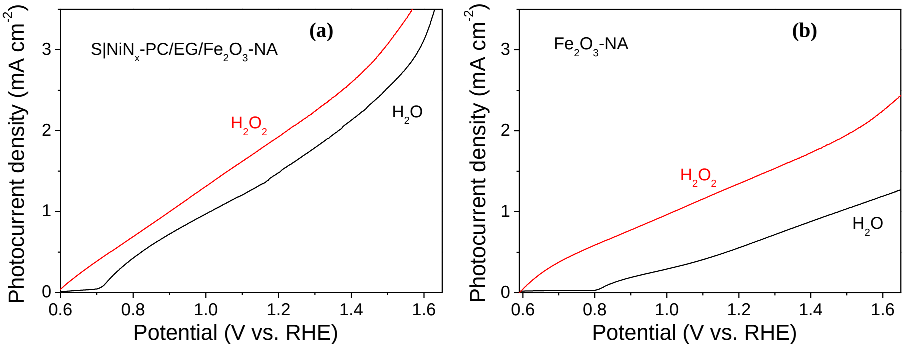

Supplementary Figure 38. **Activity comparison between two electrodes.** (a-b) Variation of photocurrent density versus applied voltage for S|NiN$_x$−PC/EG/Fe_2O_3-NA and Fe_2O_3-NA under AM 1.5G irradiation with H_2O_2 or H_2O added into the 1.0 M NaOH.

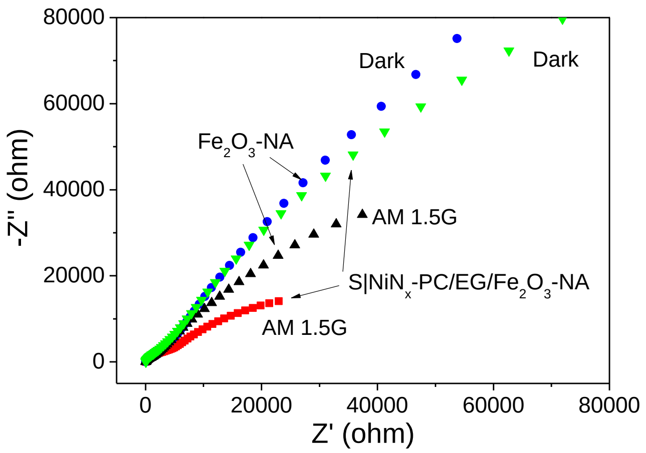

Supplementary Figure 39. **EIS activity comparison between two electrodes.** EIS Nyquist plots of S|NiN$_x$–PC/EG/Fe_2O_3-NA and Fe_2O_3-NA at 0.9 V in dark and under AM 1.5G irradiation in 1.0 M NaOH.

A smaller arc radius was observed for S|NiN$_x$–PC/EG/Fe_2O_3-NA both in dark and under AM 1.5G irradiation, than that of Fe_2O_3-NA, indicating that the incorporation of S|NiN$_x$–PC/EG significantly increased the interfacial electron transfer.

The PEC performance of S|NiN$_x$–PC/EG/Fe_2O_3-NA is determined by three fundamental processes involved in PEC-OER reaction, including light absorption ($\eta_{absorption}$), charge transport ($\eta_{transport}$), and charge transfer ($\eta_{transfer}$).[5-9] The practical PEC-OER photocurrent of S|NiN$_x$–PC/EG/Fe_2O_3-NA can be expressed as $J_{H2O} = J_{max} \times \eta_{absorption} \times \eta_{transport} \times \eta_{transfer}$.[10, 11] The J_{max} is determined by its band gap energy.[12] The $\eta_{absorption}$ is determined by integrating the light absorption over the AM 1.5G solar spectrum.[10] The $\eta_{transport}$ and $\eta_{transfer}$ are determined by comparing its PEC-OER and H_2O_2 (hole scavenger) oxidation performances.[13] Due to the fast oxidation kinetics of H_2O_2,[5] it can be assumed that the $\eta_{transfer}$ of photoelectrode is nearly 100%, resulting in the J_{H2O2} to be given by $J_{H2O2} \approx J_{max} \times \eta_{absorption} \times \eta_{transport}$, where J_{H2O2} is the photocurrent density for H_2O_2 oxidation. The value of $\eta_{transport}$ can be calculated from $\eta_{transport} \approx J_{H2O2}/(J_{max} \times \eta_{absorption})$, and the value of $\eta_{transfer}$ can be obtained by dividing J_{H2O}/J_{H2O2}. Both S|NiN$_x$–PC/EG/Fe_2O_3-NA ($\eta_{absorption}$ = 70.6%) and Fe_2O_3-NA ($\eta_{absorption}$ = 67.9%) show comparable light absorption over 300-650 nm, indicating that the coated S|NiN$_x$–PC/EG has little influence on the light absorption of Fe_2O_3-NA (Supplementary Figure 36).

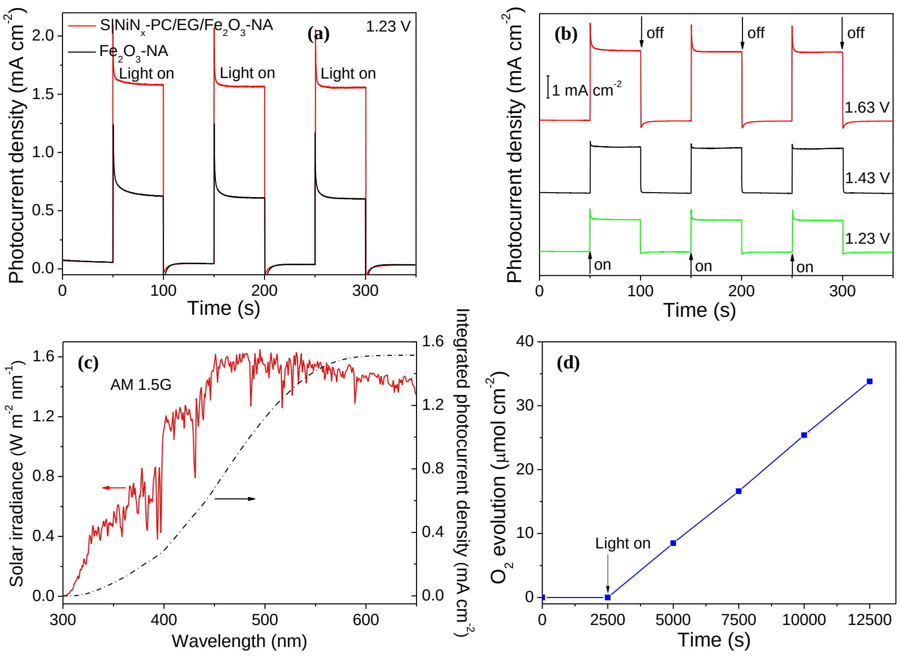

Supplementary Figure 40. **PEC-OER performance.** (a) Transient photocurrent densities vs. time of S|NiN_x−PC/EG/Fe_2O_3-NA and Fe_2O_3-NA at 1.23 V under AM 1.5G irradiation in 1.0 M NaOH. (b) Photocurrent densities vs. time of S|NiN_x−PC/EG/Fe_2O_3-NA being applied with different bias potentials under AM 1.5G irradiation in 1.0 M NaOH. (c) Solar spectral irradiance of standard AM 1.5G spectral irradiance and integrated photocurrent density of S|NiN_x−PC/EG/Fe_2O_3-NA based on the IPCE data at 1.23 V in 1.0 M NaOH. (d) Actual amounts of O_2 gas produced from the S|NiN_x−PC/EG/Fe_2O_3-NA at 1.23 V under AM 1.5G irradiation in 1.0 M NaOH.

The photocurrent density of S|NiN_x−PC/EG/Fe_2O_3-NA can be calculated by integrating the obtained IPCE spectra (Figure 5c) with standard AM 1.5G solar spectrum, using below equation:

$$J = \int_{300}^{500} \lambda \times IPCE(\lambda) \times E(\lambda) \frac{1}{1240} \times d(\lambda) \qquad 1$$

where IPCE (λ) is the obtained IPCE profile, and E (λ) is the solar irradiance at a specific wavelength (λ). Based on the IPCE data, the integrated photocurrent density of S|NiN_x−PC/EG/Fe_2O_3-NA was calculated to be 1.52 mA cm^{-2} at 1.23 V (Supplementary Figure 40c), which closely matches the value of 1.58 mA cm^{-2} measured from Figure 5. The Faradaic efficiency of S|NiN_x−PC/EG/Fe_2O_3-NA was calculated to be 95.2% (Supplementary Figure 40d), which indicates that the S|NiN_x−PC/EG/Fe_2O_3-NA is indeed PEC oxidizing water into O_2 gas.

Supplementary Tables

Supplementary Table 1. Comparison of the OER performances of different transition-metals and/or heteroatom doped carbon electrocatalysts.

Author	Catalyst (Loading density ($mg\ cm^{-2}$))[a]	Current density (J)	Potential at the corresponding J[b]	Electrolyte
**This work**	**S|NiN$_x$–PC/EG (0.15 mg cm^{-2})**	**10 mA cm^{-2}**	**1.51 V**	**1.0 M KOH**
		**100 mA cm^{-2}**	**1.56 V**	
Nat. Commun. 2013, 4, 2390	N-doped carbon (0.2 mg cm^{-2})	10 mA cm^{-2}	1.61 V	0.1 M KOH
Nat. Energy, 2016, 1, 15006	NCNTFs (0.2 mg cm^{-2})	10 mA cm^{-2}	1.60 V	0.1 M KOH
Nat. Nanotechnol. 2015, 10, 444	N,P-doped carbon foam (0.15 mg cm^{-2})	10 mA cm^{-2}	~1.88 V	0.1 M KOH
Sci. Adv. 2016, 2, e1501122	N-GRW (0.5 mg cm^{-2})	10 mA cm^{-2}	1.59 V	1.0 M KOH
Nat. Catal. 2018, 1, 63	Ni–NHGF (0.275 mg cm^{-2})	10 mA cm^{-2}	1.56 V	1.0 M KOH
Angew. Chem. Int. Ed. 2018, 57, 1856	FeCo-N$_x$-CN (0.1 mg cm^{-2})	10 mA cm^{-2}	1.60 V	1.0 M KOH
Adv. Mater. 2018, 1707319	LIG-O (2.0 mg cm^{-2})	10 mA cm^{-2}	1.59 V	1.0 M KOH
Adv. Mater. 2017, 29, 1604480	Co–N$_x$|P-GC/FEG (0.12 mg cm^{-2})	10 mA cm^{-2}	1.55 V	1.0 M KOH
Adv. Mater. 2017, 29, 1604942	SHG (2.0 mg cm^{-2})	10 mA cm^{-2}	1.56 V	0.1 M KOH
Energy Environ. Sci., 2017, 10, 2312	PEMAc@CNTs90 (0.3 mg cm^{-2})	10 mA cm^{-2}	1.53 V	1.0 M KOH

Angew. Chem. Int. Ed. 2017, 56, 610	S,N-Fe/N/C-CNT (0.6 mg cm^{-2})	10 mA cm^{-2}	1.60 V	0.1 M KOH
Adv. Mater. 2018, 30, 1705431	C-MOF-C2-900 (0.2 mg cm^{-2})	10 mA cm^{-2}	1.58 V	0.1 M KOH
J. Am. Chem. Soc. 2015, 137, 2901	echo-MWCNTs (0.2 mg cm^{-2})	10 mA cm^{-2}	1.68 V	1.0 M KOH
Angew. Chem. Int. Ed. 2014, 53, 7281	g-C_3N_4 NS–CNT (0.2 mg cm^{-2})	10 mA cm^{-2}	1.60 V	0.1 M KOH
Adv. Mater. 2016, 28, 9532	Defect graphene (0.283 mg cm^{-2})	10 mA cm^{-2}	1.57 V	1.0 M KOH
Adv. Mater. 2016, 28, 3000	NCNF-1000 (0.1 mg cm^{-2})	10 mA cm^{-2}	~1.88 V	0.1 M KOH
Adv. Mater. 2017, 29, 1606207	P-CC (---)	10 mA cm^{-2}	1.68 V	1.0 M KOH
Angew. Chem. Int. Ed. 2018, 57, 3514	CoSSPIL/CNT (0.05 mg cm^{-2})	10 mA cm^{-2}	1.64 V	0.1 M KOH
Energy Environ. Sci. 2016, 9, 1210	ONPPGC/OCC (0.1 mg cm^{-2})	10 mA cm^{-2}	1.64 V	1.0 M KOH
Adv. Mater. 2017, 29, 1703185	NGM-Co (0.5 mg cm^{-2})	10 mA cm^{-2}	~1.75 V	0.1 M KOH
Energy Environ. Sci., 2017, 10, 1186	PNGF(op) (0.1 mg cm^{-2})	10 mA cm^{-2}	~1.55 V	0.1 M KOH
Energy Environ. Sci. 2016, 9, 1661	Co_xZn_{100-x}/ZIF-8 (0.28 mg cm^{-2})	10 mA cm^{-2}	1.67 V	0.1 M KOH
Angew. Chem. Int. Ed. 2016, 55, 13296	GO-PANi31-FP (0.5 mg cm^{-2})	10 mA cm^{-2}	~1.80 V	0.1 M KOH
Energy Environ. Sci., 2016, 9, 3079	NCMT-1000 (3D) (0.82 mg cm^{-2})	10 mA cm^{-2}	1.52 V	0.1 M KOH
Angew. Chem. Int. Ed.	PCN–CFP	10 mA cm^{-2}	1.63 V	0.1 M KOH

2015, 54, 4646	(0.2 mg cm^{-2})			
J. Am. Chem. Soc. 2017, 139, 3336	Co−C_3N_4/CNT (2.0 mg cm^{-2})	10 mA cm^{-2}	1.61 V	1.0 M KOH
Adv. Funct. Mater. 2015, 25, 872	N/Co-doped PCP//NRGO (0.714 mg cm^{-2})	10 mA cm^{-2}	1.66 V	0.1 M KOH
Adv. Energy Mater. 2016, 6, 1501794	GNS/MC (1.21 mg cm^{-2})	10 mA cm^{-2}	1.57 V	0.1 M KOH
Adv. Energy Mater. 2016, 6, 1501492	NSGF ---	10 mA cm^{-2}	1.58 V	1.0 M KOH
Adv. Energy Mater. 2015, 5, 1401660	Ni@NC (0.4 mg cm^{-2})	10 mA cm^{-2}	1.62 V	0.1 M KOH
Adv. Energy Mater. 2016, 6, 1501966	S,S'-CNT_{1000} (0.23 mg cm^{-2})	10 mA cm^{-2}	1.58 V	1.0 M KOH
Adv. Energy Mater. 2016, 1502039	NH_2-CDs-3 (0.014 mg cm^{-2})	10 mA cm^{-2}	~1.55 V	0.1 M KOH
Adv. Energy Mater. 2016, 6, 1501966	S,S′-CNT 1000 ^oC (0.23 mg cm^{-2})	10 mA cm^{-2}	~1.58 V	1.0 M KOH
Adv. Energy Mater. 2017, 7, 1602068	N,S-CNT ---	10 mA cm^{-2}	~1.59 V	1.0 M KOH
Adv. Funct. Mater. 2017, 27, 1606497	PPy/FeTCPP/Co (0.3 mg cm^{-2})	10 mA cm^{-2}	1.61 V	0.1 M KOH
Adv. Energy Mater. 2017, 7, 1602928	NPC-CP (0.6 mg cm^{-2})	10 mA cm^{-2}	1.54 V	1.0 M KOH
Nano Energy 2016, 30, 503	N-MGF (1.0 mg cm^{-2})	10 mA cm^{-2}	1.55 V	0.1 M KOH
Small 2014, 10, 2251	NGSH (0.26 mg cm^{-2})	10 mA cm^{-2}	1.63 V	0.1 M KOH
Adv. Sci. 2015, 2,	G-C_3N_4	100 mA cm^{-2}	>1.80 V	0.1 M KOH

1400015	(0.1 mg cm^{-2})			
Energy Storage Materials, 2015, 1, 17	NSCG (0.2 mg cm^{-2})	100 mA cm^{-2}	~1.89 V	0.1 M KOH

[a] A high mass-loading is of great significance for high catalytic performance and real application.[14-16]

[b] All potentials were converted to the reversible hydrogen electrode (RHE) scale, unless otherwise stated.

Supplementary Table 2. Comparison of the TOF values of other reported non-noble metal OER electrocatalysts.

Catalyst	Potential	TOF (s^{-1})
**S|NiN$_x$–PC/EG/ (this work)**	**1.53 V**	**10.9**
Amorphous Ni-FeO$_x$[17]	1.53 V	0.21
Ni-Fe LDH/CNT[18]	1.53 V	0.56
Ni-FeO$_x$ (solution-cast)[19]	1.53 V	0.06
$Ni_{0.75}V_{0.25}$-LDH[20]	1.58 V	0.054
CoV_2O_6–V_2O_5/NRGO[21]	1.53 V	1.8
Hierarchical $CoTe_2$[22]	1.58 V	0.2
NiFe-MOF[23]	1.63 V	3.8
$Fe(PO_3)_2$/Ni_2P[24]	1.53 V	0.12
CoFe LDHs[25]	1.53 V	4.78
Fe_3-Co_2@Ni[26]	1.53 V	1.82
Co/Co_2P[27]	1.65 V	0.11
$Fe_{0.5}V_{0.5}$[28]	1.63 V	0.02
Fe_1Co_1-ONS[29]	1.58 V	0.022

Supplementary Table 3. Comparison of the PEC-OER performances of other reported Fe_2O_3-based photoanodes.

Catalyst	Photocurrent density (J) at 1.23 V	Potential	Electrolyte	Light source
**S|NiN$_x$−PC/EG/Fe_2O_3-NA (this work)**	**1.58 mA cm^{-2}**	**1.23 V**	**1.0 M NaOH**	**200 W Xenon lamp (AM 1.5G) I_0 = 100 mW cm^{-2}**
Porous α-Fe_2O_3 NWs[30]	0.54 mA cm^{-2}	1.23 V	1.0 M NaOH	1000 W Xenon lamp (AM 1.5G) I_0 = 100 mW cm^{-2}
Fe_2O_3/Fe_2TiO_5[31]	1.63 mA cm^{-2}	1.23 V	1.0 M KOH	300 W Xenon lamp (AM 1.5G) I_0 = 100 mW cm^{-2}
Co_3O_4/Fe_2O_3[32]	1.20 mA cm^{-2}	1.23 V	1.0 M NaOH	150 W Xenon lamp (AM 1.5G) I_0 = 100 mW cm^{-2}
Fe-Pi/Fe_2O_3[33]	～0.80 mA cm^{-2}	1.23 V	1.0 M NaOH	300 W Xenon lamp (AM 1.5G) I_0 = 100 mW cm^{-2}
Fe_2O_3/NiOOH[34]	0.625 mA cm^{-2}	1.23 V	1.0 M NaOH	300 W Xenon lamp (AM 1.5G) I_0 = 100 mW cm^{-2}
FeOOH/M:B-Fe_2O_3[35]	2.35 mA cm^{-2}	1.23 V	1.0 M NaOH	Xenon lamp (AM 1.5G) I_0 = 100 mW cm^{-2}
Ni:FeOOH/Fe_2O_3[36]	～1.40 mA cm^{-2}	1.23 V	1.0 M NaOH	Xenon lamp (AM 1.5G) I_0 = 100 mW cm^{-2}
Fe_2TiO_5/Fe_2O_3/Pt[37]	～1.0 mA cm^{-2}	1.23 V	1.0 M KOH	150 W Xenon lamp

				(AM 1.5G) I_0 = 100 mW cm^{-2}
Co-Pi/Ag/Fe_2O_3[38]	4.68 mA cm^{-2}	1.23 V	1.0 M NaOH	Xenon lamp (AM 1.5G) I_0 = 100 mW cm^{-2}
FeOOH/Fe_2O_3[39]	～1.3 mA cm^{-2}	1.23 V	1.0 M NaOH	Xenon lamp (AM 1.5G) I_0 = 100 mW cm^{-2}
Zr-Fe_2O_3 NT[40]	1.5 mA cm^{-2}	1.23 V	1.0 M KOH	300 W Xenon lamp (AM 1.5G) I_0 = 100 mW cm^{-2}
C/Co_3O_4-Fe_2O_3[41]	1.48 mA cm^{-2}	1.23 V	1.0 M NaOH	300 W Xenon lamp (AM 1.5G) I_0 = 100 mW cm^{-2}
Ti-(SiO_x/np-Fe_2O_3)[42]	2.44 mA cm^{-2}	1.23 V	1.0 M NaOH	Xenon lamp (AM 1.5G) I_0 = 100 mW cm^{-2}
TiO_2/Ti:Fe_2O_3 BNR[43]	2.5 mA cm^{-2}	1.23 V	1.0 M KOH	300 W Xenon lamp (AM 1.5G) I_0 = 100 mW cm^{-2}

Supplementary Methods

Synthesis of NiN_x−PC/EG

The EG was immersed into 20 mL mixture solution of 0.15 g $NiCl_2•6H_2O$ and 0.5 g dicyanamide, and then, they were transferred into a Teflon-lined autoclave for hydrothermal reaction at 200 ℃ for 4 h. Finally, the obtained DN/EG electrode was pyrolyzed at 900 ℃ under flowing Ar atmosphere for 3 h, followed by acid etching treatment with 0.5 M H_2SO_4 to remove unstable nickel species. The loading amount of NiN_x−PC/EG on graphite foil was ~ 0.13 mg cm^{-2}.

Synthesis of Ni-S−PC/EG

The EG was immersed into 20 mL mixture solution of 0.15 g $NiCl_2•6H_2O$ and 0.5 mL thiophene under stirring, and then, they were transferred into a Teflon-lined autoclave for hydrothermal reaction at 200 ℃ for 4 h. Finally, the obtained TN/EG electrode was pyrolyzed at 900 ℃ under flowing Ar atmosphere for 3 h, followed by acid etching treatment with 0.5 M H_2SO_4 to remove unstable nickel species. The loading amount of Ni-S−PC/EG on graphite foil was ~ 0.11 mg cm^{-2}.

Synthesis of N-S−PC/EG

The EG was immersed into 20 mL mixture solution of 0.5 g dicyanamide and 0.5 mL thiophene under stirring, and then, they were transferred into a Teflon-lined autoclave for hydrothermal reaction at 200 ℃ for 4 h. Finally, the obtained DT/EG electrode was pyrolyzed at 900 ℃ under flowing Ar atmosphere for 3 h, followed by acid etching treatment with 0.5 M H_2SO_4 to remove unstable nickel species. The loading amount of N-S−PC/EG on graphite foil was ~ 0.08 mg cm^{-2}.

Synthesis of S|NiN_x−PC/EG before etching

The EG was immersed into 20 mL mixture solution of 0.15 g $NiCl_2•6H_2O$, 0.5 g dicyanamide, and 0.5 mL thiophene under stirring, and then, they were transferred into a Teflon-lined autoclave for hydrothermal reaction at 200 ℃ for 4 h. Finally, the obtained TSC/EG electrode was pyrolyzed at 900 ℃ under flowing Ar atmosphere for 3 h.

Synthesis of TSC/EG

The EG was immersed into 20 mL mixture solution of 0.15 g $NiCl_2•6H_2O$, 0.5 g

dicyanamide, and 0.5 mL thiophene under stirring, and then, they were transferred into a Teflon-lined autoclave for hydrothermal reaction at 200 ℃ for 4 h.

Synthesis of S|CoN$_x$−PC/EG

The EG was immersed into 20 mL mixture solution of 0.18 g $Co(NO_3)_2$•$6H_2O$, 0.5 g dicyanamide, and 0.5 mL thiophene under stirring, and then, they were transferred into a Teflon-lined autoclave for hydrothermal reaction at 200 ℃ for 4 h. Finally, the obtained TSC/EG electrode was pyrolyzed at 900 ℃ under flowing Ar atmosphere for 3 h, followed by acid etching treatment with 0.5 M H_2SO_4 to remove unstable cobalt species.

Synthesis of S|FeN$_x$−PC/EG

The EG was immersed into 20 mL mixture solution of 0.25 g $Fe(NO_3)_3$•$9H_2O$, 0.5 g dicyanamide, and 0.5 mL thiophene under stirring, and then, they were transferred into a Teflon-lined autoclave for hydrothermal reaction at 200 ℃ for 4 h. Finally, the obtained TSC/EG electrode was pyrolyzed at 900 ℃ under flowing Ar atmosphere for 3 h, followed by acid etching treatment with 0.5 M H_2SO_4 to remove unstable iron species.

Synthesis of S|NiN$_x$−PC

A 20 mL mixture solution of 0.15 g $NiCl_2$•$6H_2O$, 0.5 g dicyanamide, and 0.5 mL thiophene was stirred for 2 h and then added into a Teflon-lined autoclave for hydrothermal reaction at 200 ℃ for 4 h. The obtained TSC powder was pyrolyzed at 900 ℃ under flowing Ar atmosphere for 3 h, followed by acid etching treatment with 0.5 M H_2SO_4 to remove unstable nickel species.

Synthesis of S|NiN$_x$−PC/EG-1/4Ni

The EG was immersed into 20 mL mixture solution of 0.0375 g $NiCl_2$•$6H_2O$, 0.5 g dicyanamide, and 0.5 mL thiophene under stirring, and then, they were transferred into a Teflon-lined autoclave for hydrothermal reaction at 200 ℃ for 4 h. Finally, the obtained TSC/EG electrode was pyrolyzed at 900 ℃ under flowing Ar atmosphere for 3 h, followed by acid etching treatment with 0.5 M H_2SO_4 to remove unstable nickel species.

Synthesis of S|NiN$_x$−PC/EG-1/2Ni

The EG was immersed into 20 mL mixture solution of 0.075 g $NiCl_2•6H_2O$, 0.5 g dicyanamide, and 0.5 mL thiophene under stirring, and then, they were transferred into a Teflon-lined autoclave for hydrothermal reaction at 200 ℃ for 4 h. Finally, the obtained TSC/EG electrode was pyrolyzed at 900 ℃ under flowing Ar atmosphere for 3 h, followed by acid etching treatment with 0.5 M H_2SO_4 to remove unstable nickel species.

Synthesis of S|NiN$_x$−PC/EG-2/1Ni

The EG was immersed into 20 mL mixture solution of 0.3 g $NiCl_2•6H_2O$, 0.5 g dicyanamide, and 0.5 mL thiophene under stirring, and then, they were transferred into a Teflon-lined autoclave for hydrothermal reaction at 200 ℃ for 4 h. Finally, the obtained TSC/EG electrode was pyrolyzed at 900 ℃ under flowing Ar atmosphere for 3 h, followed by acid etching treatment with 0.5 M H_2SO_4 to remove unstable nickel species.

Synthesis of S|NiN$_x$−PC/EG (700 ^{o}C)

The EG was immersed into 20 mL mixture solution of 0.15 g $NiCl_2•6H_2O$, 0.5 g dicyanamide, and 0.5 mL thiophene under stirring, and then, they were transferred into a Teflon-lined autoclave for hydrothermal reaction at 200 ℃ for 4 h. Finally, the obtained TSC/EG electrode was pyrolyzed at 700 ℃ under flowing Ar atmosphere for 3 h, followed by acid etching treatment with 0.5 M H_2SO_4 to remove unstable nickel species. The loading amount of S|NiN$_x$−PC/EG (700 ^{o}C) on graphite foil was ~ 0.18 mg cm^{-2}.

Synthesis of S|NiN$_x$−PC/EG (800 ^{o}C)

The EG was immersed into 20 mL mixture solution of 0.15 g $NiCl_2•6H_2O$, 0.5 g dicyanamide, and 0.5 mL thiophene under stirring, and then, they were transferred into a Teflon-lined autoclave for hydrothermal reaction at 200 ℃ for 4 h. Finally, the obtained TSC/EG electrode was pyrolyzed at 800 ℃ under flowing Ar atmosphere for 3 h, followed by acid etching treatment with 0.5 M H_2SO_4 to remove unstable nickel species. The loading amount of S|NiN$_x$−PC/EG (800 ^{o}C) on graphite foil was ~ 0.17 mg cm^{-2}.

Synthesis of S|NiN$_x$−PC/EG (1000 ^{o}C)

The EG was immersed into 20 mL mixture solution of 0.15 g $NiCl_2$•$6H_2O$, 0.5 g dicyanamide, and 0.5 mL thiophene under stirring, and then, they were transferred into a Teflon-lined autoclave for hydrothermal reaction at 200 ℃ for 4 h. Finally, the obtained TSC/EG electrode was pyrolyzed at 1,000 ℃ under flowing Ar atmosphere for 3 h, followed by acid etching treatment with 0.5 M H_2SO_4 to remove unstable nickel species. The loading amount of S|NiN_x−PC/EG (1,000 ^oC) on graphite foil was ~ 0.12 mg cm^{-2}.

Synthesis of Fe_2O_3-NA

The FTO substrate was placed in an autoclave with a 20 mL aqueous solution containing 0.27 g of $FeCl_3$•$6H_2O$ and 0.14 g of Na_2SO_4.[44] The Teflon-lined autoclave was subsequently heated at 120 ℃ for 6 h. The resulting product was further allowed to cool to room temperature, and collected and rinsed with ethanol and deionized water separately. Finally, Fe_2O_3-NA was obtained from the as-prepared catalyst in a tube furnace at 500 ℃ with a ramp rate of 2 ℃ min^{-1} for 3 h under Air.

Synthesis of S|NiN_x−PC/EG/Fe_2O_3-NA

The obtained S|NiN_x−PC/EG powder (5 mg) was suspended in isopropyl alcohol (5 mL) and spin-coated onto the Fe_2O_3-NA surface at 2,000 rpm for 20 s. The sample was then annealed at 100 ℃ for 20 min. The spin coating and calcination steps were repeated three times to achieve desired loading.

Characterization

The prepared EG-based electrodes were carefully scraped off from graphite foil and used for XRD, FTIR, TEM, NMR, EDX, ICP-OES, and N_2 adsorption measurements. For XPS, contact angle, FESEM, and Raman, the catalysts grown on graphite foil were measured directly.

Electrochemical measurements

The reference was calibrated against and converted to eversible hydrogen electrode (RHE) according to the Nernst equation ($E_{RHE} = E_{Ag/Ag/Cl} + 0.059 \times pH + 0.197$). Mass activity (mA mg^{-1}) was calculated from the catalyst loading m (mg cm^{-2}) and the measured current density J (mA cm^{-2}) at 1.58 V according to the formula: Mass activity = J/m. For comparison, 20 mg of Ir/C was dispersed in 0.2 mL of a 0.5 wt.%

Nafion solution and 0.8 mL of ethanol under an ultra-sonication. The dispersion was then dropped on the surface of the graphite foil, yielding an approximate catalyst loading of 0.15 mg cm^{-2}, the same as that of S|NiN_x−PC/EG on graphite foil. Unless otherwise noted, all polarization curves were iR corrected. The turnover frequency (TOF) of S|NiN_x−PC/EG catalyst was calculated according to the previous reports.[20]
In contrast, the OER activity of powder S|NiN_x−PC/EG obtained by scratching down the S|NiN_x−PC/EG from graphite foil and then deposited on glassy carbon electrode was evaluated by using the rotating disk electrode (RDE) technique at a rotation rate of 1600 rpm in 1.0 M KOH solution.[45]

PEC measurements

Considering that back-illumination usually suffer from unproductive light absorption from FTO substrate, the PEC measurements were conducted under front-side illumination in all cases (light enters from the absorber side).[36, 46] The stability of the photoanodes was evaluated by measuring the photocurrent density versus time curves at 1.23 V. The charge transfer efficiencies were estimated as functions of applied potential by using H_2O_2 as a hole scavenger under AM 1.5G irradiation. We assume that the oxidation kinetics of H_2O_2 is very fast and its charge transfer efficiency is 100%. Therefore, the ratio of photocurrent densities measured in H_2O and H_2O_2 gives arise to the charge-transfer efficiency.[10]
Photoconversion efficiency (η), which is the light energy to chemical energy conversion efficiency, is calculated as

$$\eta = [1.23 - V_{app}) \times (J_{light} - J_{dark})/P_{light}] \times 100\% \quad 2$$

where V_{app} is the applied potential versus RHE, J_{dark} and J_{light} are the respective current densities in dark and under AM 1.5 G irradiation, and P_{light} is the power density of AM 1.5G (100 mW cm^{-2}). The EIS was performed at a DC bias of 0.9 V in the frequency range of 100 K−0.01 Hz with an AC voltage of 10 mV.

Computational details

All first-principles DFT calculations about the OER theoretical mechanisms were performed by using the Cambridge Serial Total Energy Package (CASTEP) in Material Studio that is based on the DFT plane-wave norm-conserving

pseudopotential approach. For calculation of electronic structures, kinetics performance and catalytic reactions, the electron structures were conducted with the CASTEP in Material Studio, which employs the DFT plane-wave norm-conserving pseudopotential method to conduct first principles quantum mechanics calculations. In our simulations, the generalized gradient approximation (GGA) within Perdew–Burke–Ernzerhof (PBE) form was chosen as the exchange–correlation function. The convergence tests of the total energy with respect to the *k*-points sampling and the energy-cutoff were carefully examined, using 1 × 1 × 5 Monkhorst-Pack *k*-points grid and a 500 eV energy cut-off for plane-wave expansion. Valence states used were Ni-$3s^2 3p^6 3d^8 4s^2$, C-$2s^2 2p^2$, N-$2s^2 2p^3$, O-$2s^2 2p^4$, S-$3s^2 3p^4$, and H-$1s^1$. The N–S, Ni–S, Ni–N_4, and Ni–N_3S were doped on the armchair (4, 0) and zigzag (3, 3) graphene nanoribbons, at both edges of which the un-saturated dangling bonds were terminated with H atoms. In the supercell configuration, we maintained a sufficiently large separation (20 Å) along the *y*-axis. The spin polarization and formal spin as initial was set to all the DFT calculations. The SCF tolerance was set to 5e-6eV atom^{-1} for the geometrical optimization and 5e-7eV atom^{-1} for the electronic structures and correlative phonon calculations.

To model the thermochemistry mechanism of OER elementary steps, the following four electrons reaction pathways is the most convenient way to proceed:

$$H_2O(l) + \otimes \rightarrow OH^{\otimes} + (H^+ + e^-) \quad 3$$

$$OH^{\otimes} \rightarrow O^{\otimes} + (H^+ + e^-) \quad 4$$

$$O^{\otimes} + H_2O(l) \rightarrow OOH^{\otimes} + (H^+ + e^-) \quad 5$$

$$OOH^{\otimes} \rightarrow \otimes + O_2(g) + (H^+ + e^-) \quad 6$$

in these equations, the $\otimes$ is an active site on the catalyst surface; (l) and (g) represent the liquid and gas phases of H_2O and O_2, respectively; the $O^{\otimes}$, $OH^{\otimes}$, and $OOH^{\otimes}$ are adsorbed reaction intermediates with groups O^-, OH^-, and OOH^-, respectively. To perform the simulations, the absorption energies can be expressed as the following:

$$\Delta E_{OH^{\otimes}} = E(OH^{\otimes}) - E(\otimes) - (E_{H_2O} - 1/2E_{H_2}) \quad 7$$

$$\Delta E_{OOH^{\otimes}} = E(OOH^{\otimes}) - E(\otimes) - (2E_{H_2O} - 3/2E_{H_2}) \quad 8$$

$$\Delta E_{O^{\otimes}} = E(O^{\otimes}) - E(\otimes) - (E_{H_2O} - E_{H_2}) \quad 9$$

In equations S5-S7, the $E(\otimes)$ is the ground-state energy of material surface without groups; those energies of the catalyst surfaces adsorbed with groups O^-, OH^-, and OOH^- are $E(O^{\otimes})$, $E(OH^{\otimes})$, and $E(OOH^{\otimes})$, respectively. The zero point energy (ZPE) and entropy corrections are involved in the absorption energies for the free energies of adsorption which the equation can be expressed as follows,

$$\Delta G_{ads} = \Delta E_{ads} + \Delta ZPE - T\Delta S \quad 10$$

The reaction free energy for each elementary reaction step is given by:

$$\Delta G = \Delta E + \Delta ZPE - T\Delta S + \Delta G_U + \Delta G_{PH} \quad 11$$

The ΔE is the reaction energies difference between reactant and product; ΔG_U is obtained from the product of the potential of the electrode *V* and the electron charge *e*; ΔG_{PH} is the Nernst equation as follows,

$$\Delta G_{PH} = -k_B T \ln[H^+] \quad 12$$

The zero-point energy change ΔZPE can be expressed as vibrational frequency calculation:

$$\Delta ZPE = 1/2\sum hv_i \quad 13$$

The v_i is the vibrational frequencies.

For the OER processes, the reaction free energies obtained in Equations S1 through S4 were calculated by using Equation S9. Furthermore, the overpotentials can be definition by Nørskov *et al.*,[2] and are then readily expressed as:

$$G^{OER} = \max\{\Delta G_1, \Delta G_2, \Delta G_3, \Delta G_4\} \quad 14$$

$$\eta^{OER} = G^{OER}/e - 1.23V \quad 15$$

We note that the ΔG_1, ΔG_2, ΔG_3, and ΔG_4 are, respectively, the reaction free

energies given in Equations S1-S4. The overpotential is only a thermodynamic quantity and they have been found to scale well with the experiment results.

Supplementary References

1. Hou, Y., Huang, T., Wen, Z., Mao, S., Cui, S. & Chen, J. Metal−organic framework-derived nitrogen-doped core-shell-structured porous Fe/Fe_3C@C nanoboxes supported on graphene sheets for efficient oxygen reduction reactions. *Adv. Energy Mater.* **4**, 1400337 (2014).
2. Jia, Y., *et al.* A heterostructure coupling of exfoliated Ni–Fe hydroxide nanosheet and defective graphene as a bifunctional electrocatalyst for overall water splitting. *Adv. Mater.* **29**, 1700017 (2017).
3. Hou, Y., *et al.* Efficient electrochemical and photoelectrochemical water splitting by a 3D nanostructured carbon supported on flexible exfoliated graphene foil. *Adv. Mater.* **29**, 1604480 (2017).
4. Kong, D., Wang, H., Lu, Z. & Cui, Y. $CoSe_2$ nanoparticles grown on carbon fiber paper: an efficient and stable electrocatalyst for hydrogen evolution reaction. *J. Am. Chem. Soc.* **136**, 4897-4900 (2014).
5. Cho, I. S., *et al.* Codoping titanium dioxide nanowires with tungsten and carbon for enhanced photoelectrochemical performance. *Nat. Commun.* **4**, 1723 (2013).
6. Zhou, L., *et al.* High light absorption and charge separation efficiency at low applied voltage from Sb-doped SnO_2/$BiVO_4$ core/shell nanorod-array photoanodes. *Nano Lett.* **16**, 3463-3474 (2016).
7. Huang, J. W., Zhang, Y. & Ding, Y. Rationally designed/constructed CoO_x/WO_3 anode for efficient photoelectrochemical water oxidation. *ACS Catal.* **7**, 1841-1845 (2017).
8. Ma, M., Zhang, K., Li, P., Jung, M. S., Jeong, M. J. & Park, J. H. Dual oxygen and tungsten vacancies on a WO_3 photoanode for enhanced water oxidation. *Angew. Chem. Int. Ed.* **55**, 11819-11823 (2016).
9. Kim, T. W. & Choi, K. S. Nanoporous $BiVO_4$ photoanodes with dual-layer oxygen evolution catalysts for solar water splitting. *Science* **343**, 990-994 (2014).
10. Rao, P. M., *et al.* Simultaneously efficient light absorption and charge

separation in WO_3/$BiVO_4$ core/shell nanowire photoanode for photoelectrochemical water oxidation. *Nano Lett.* **14**, 1099-1105 (2014).

11. Shi, X., *et al.* Efficient photoelectrochemical hydrogen production from bismuth vanadate-decorated tungsten trioxide helix nanostructures. *Nat. Commun.* **5**, 4775 (2014).

12. Zhang, R., *et al.* Enhanced photoelectrochemical water oxidation performance of Fe_2O_3 nanorods array by S doping. *ACS Sustain. Chem. Eng.* **5**, 7502-7506 (2017).

13. Shin, S., *et al.* A tree-like nanoporous WO_3 photoanode with enhanced charge transport efficiency for photoelectrochemical water oxidation. *J. Mater. Chem., A* **3**, 12920-12926 (2015).

14. Zheng, Y. R., Gao, M. R., Yu, Z. Y., Gao, Q., Gao, H. L. & Yu, S. H. Cobalt diselenide nanobelts grafted on carbon fiber felt: an efficient and robust 3D cathode for hydrogen production. *Chem. Sci.* **6**, 4594-4598 (2015).

15. Wang, D. Y., *et al.* Highly active and stable hybrid catalyst of cobalt-doped FeS_2 nanosheets-carbon nanotubes for hydrogen evolution reaction. *J. Am. Chem. Soc.* **137**, 1587-1592 (2015).

16. Tian, J., Liu, Q., Cheng, N., Asiri, A. M. & Sun, X. Self-supported Cu_3P nanowire arrays as an integrated high-performance three-dimensional cathode for generating hydrogen from water. *Angew. Chem. Int. Ed.* **53**, 9577-9581 (2014).

17. Qiu, Y., Xin, L. & Li, W. Electrocatalytic oxygen evolution over supported small amorphous Ni-Fe nanoparticles in alkaline electrolyte. *Langmuir* **30**, 7893-7901 (2014).

18. Gong, M., *et al.* An advanced Ni-Fe layered double hydroxide electrocatalyst for water oxidation. *J. Am. Chem. Soc.* **135**, 8452-8455 (2013).

19. Trotochaud, L., Ranney, J. K., Williams, K. N. & Boettcher, S. W. Solution-cast metal oxide thin film electrocatalysts for oxygen evolution. *J. Am. Chem. Soc.* **134**, 17253-17261 (2012).

20. Fan, K., *et al.* Nickel–vanadium monolayer double hydroxide for efficient

electrochemical water oxidation. *Nat. Commun.* **7**, 11981 (2016).

21. Shen, F. C., *et al.* CoV_2O_6–V_2O_5 coupled with porous N-doped reduced graphene oxide composite as a highly efficient electrocatalyst for oxygen evolution. *ACS Energy Lett.* **2**, 1327-1333 (2017).

22. Gao, Q., *et al.* Phase-selective syntheses of cobalt telluride nanofleeces for efficient oxygen evolution catalysts. *Angew. Chem. Int. Ed.* **56**, 7769-7773 (2017).

23. Duan, J., Chen, S. & Zhao, C. Ultrathin metal-organic framework array for efficient electrocatalytic water splitting. *Nat. Commun.* **8**, 15341 (2017).

24. Zhou, H., *et al.* Highly active catalyst derived from a 3D foam of $Fe(PO_3)_2$/Ni_2P for extremely efficient water oxidation. *Proc. Natl. Acad. Sci.* **114**, 5607-5611 (2017).

25. Wang, Y., *et al.* Layered double hydroxide nanosheets with multiple vacancies obtained by dry exfoliation as highly efficient oxygen evolution electrocatalysts. *Angew. Chem. Int. Ed.* **56**, 5867-5871 (2017).

26. Shen, J. Q., *et al.* Modular and stepwise synthesis of a hybrid metal-organic framework for efficient electrocatalytic oxygen evolution. *J. Am. Chem. Soc.* **139**, 1778-1781 (2017).

27. Masa, J., *et al.* Low overpotential water splitting using cobalt–cobalt phosphide nanoparticles supported on nickel foam. *ACS Energy Lett.* **1**, 1192-1198 (2016).

28. Fan, K., *et al.* Hollow iron-vanadium composite spheres: a highly efficient iron-based water oxidation electrocatalyst without the need for nickel or cobalt. *Angew. Chem. Int. Ed.* **56**, 3289-3293 (2017).

29. Zhuang, L., *et al.* Ultrathin iron-cobalt oxide nanosheets with abundant oxygen vacancies for the oxygen evolution reaction. *Adv. Mater.* **29**, 1606793 (2017).

30. Li, L., Yu, Y., Meng, F., Tan, Y., Hamers, R. J. & Jin, S. Facile solution synthesis of α-$FeF_3 \cdot 3H_2O$ nanowires and their conversion to α-Fe_2O_3 nanowires for photoelectrochemical application. *Nano Lett.* **12**, 724-731

(2012).

31. Li, C., Wang, T., Luo, Z., Liu, S. & Gong, J. Enhanced charge separation through ALD-modified Fe_2O_3/Fe_2TiO_5 nanorod heterojunction for photoelectrochemical water oxidation. *Small* **12**, 3415-3422 (2016).

32. Xi, L., *et al.* Co_3O_4-decorated hematite nanorods as an effective photoanode for solar water oxidation. *J. Phys. Chem., C* **116**, 13884-13889 (2012).

33. Hu, Z., Shen, Z. & Yu, J. C. Covalent fixation of surface oxygen atoms on hematite photoanode for enhanced water oxidation. *Chem. Mater.* **28**, 564-572 (2016).

34. Malara, F., *et al.* α-Fe_2O_3/NiOOH: An effective heterostructure for photoelectrochemical water oxidation. *ACS Catal.* **5**, 5292-5300 (2015).

35. Ahn, H. J., Yoon, K. Y., Kwak, M. J., Park, J. & Jang, J. H. Boron doping of metal-doped hematite for reduced surface recombination in water splitting. *ACS Catal.* **8**, 11932-11939 (2018).

36. Cai, L., *et al.* One-step hydrothermal deposition of Ni:FeOOH onto photoanodes for enhanced water oxidation. *ACS Energy Lett.* **1**, 624-632 (2016).

37. Wang, L., Nguyen, N. T., Huang, X., Schmuki, P. & Bi, Y. Hematite photoanodes: synergetic enhancement of light harvesting and charge management by sandwiched with Fe_2TiO_5/Fe_2O_3/Pt structures. *Adv. Funct. Mater.* **27**, 1703527 (2017).

38. Peerakiatkhajohn, P., Yun, J. H., Chen, H., Lyu, M., Butburee, T. & Wang, L. Stable hematite nanosheet photoanodes for enhanced photoelectrochemical water splitting. *Adv. Mater.* **28**, 6405-6410 (2016).

39. Kim, J. Y., Youn, D. H., Kang, K. & Lee, J. S. Highly conformal deposition of an ultrathin FeOOH layer on a hematite nanostructure for efficient solar water splitting. *Angew. Chem. Int. Ed.* **55**, 10854-10858 (2016).

40. Li, C., *et al.* Surviving high-temperature calcination: ZrO_2-induced hematite nanotubes for photoelectrochemical water oxidation. *Angew. Chem. Int. Ed.* **56**, 4150-4155 (2017).

41. Zhang, P., Wang, T., Chang, X., Zhang, L. & Gong, J. Synergistic cocatalytic effect of carbon nanodots and Co_3O_4 nanoclusters for the photoelectrochemical water oxidation on hematite. *Angew. Chem. Int. Ed.* **55**, 5851-5855 (2016).
42. Ahn, H. J., Yoon, K. Y., Kwak, M. J. & Jang, J. H. A Titanium-doped SiO_x passivation layer for greatly enhanced performance of a hematite-based photoelectrochemical system. *Angew. Chem. Int. Ed.* **55**, 9922-9926 (2016).
43. Luo, Z., Wang, T., Zhang, J., Li, C., Li, H. & Gong, J. Dendritic hematite nanoarray photoanode modified with a conformal titanium dioxide interlayer for effective charge collection. *Angew. Chem. Int. Ed.* **56**, 12878-12882 (2017).
44. Hou, Y., Zuo, F., Dagg, A. & Feng, P. Visible light-driven alpha-Fe_2O_3 nanorod/graphene/$BiV_{1-x}Mo_xO_4$ core/shell heterojunction array for efficient photoelectrochemical water splitting. *Nano Lett.* **12**, 6464-6473 (2012).
45. Hou, Y., Cui, S., Wen, Z., Guo, X., Feng, X. & Chen, J. Strongly coupled 3D hybrids of N-doped porous carbon nanosheet/CoNi alloy-encapsulated carbon nanotubes for enhanced electrocatalysis. *Small* **11**, 5940-5948 (2015).
46. Kuang, Y., Jia, Q., Nishiyama, H., Yamada, T., Kudo, A. & Domen, K. A front-illuminated nanostructured transparent $BiVO_4$ photoanode for >2% efficient water splitting. *Adv. Energy Mater.* **6**, 1501645 (2016).
